# Total irrigation by crop in the Continental United States from 2008 to 2020

**DOI:** 10.1038/s41597-024-03244-w

**Published:** 2024-04-17

**Authors:** P. J. Ruess, Megan Konar, Niko Wanders, Marc F. P. Bierkens

**Affiliations:** 1 Civil and Environmental Engineering, University of Illinois at Urbana-Champaign, Urbana, USA; 2 Department of Physical Geography, Utrecht University, Utrecht, The Netherlands; 3 Unit Subsoil and Groundwater Systems, Deltares, Utrecht, The Netherlands

**Keywords:** Water resources, Agriculture

## Abstract

We provide a dataset of irrigation water withdrawals by crop, county, year, and water source within the United States. We employ a framework we previously developed to establish a companion dataset to our original estimates. The main difference is that we now use the U.S. Geological Survey (USGS) variable ‘irrigation — total’ to partition PCR-GLOBWB 2 hydrology model estimates, instead of ‘irrigation — crop’ as used in previous estimates. Our findings for Surface Water Withdrawals (SWW), total Groundwater Withdrawals (GWW), and nonrenewable Groundwater Depletion (GWD) are similar to those of prior estimates but now have better spatial coverage, since several states are missing from the USGS ‘irrigation — crop’ variable that was originally used. Irrigation water use increases in this study, since more states are included and ‘irrigation — total’ includes more categories of irrigation than ‘irrigation — crop’. Notably, irrigation in the Mississippi Embayment Aquifer is now captured for rice and soy. We provide nearly 2.5 million data points with this paper (3,142 counties; 13 years; 3 water sources; and 20 crops).

## Background & Summary

This paper presents a companion dataset to Ruess *et al*.^1^. Ruess *et al*.^1^ estimated Irrigation Water Use (IWU) by crop for counties in the Continental United States (CONUS) from 2008 to 2020 Ruess *et al*.^1^ separated IWU into three water source categories: Surface Water Withdrawals (SWW), Groundwater Withdrawals (GWW), and Groundwater Depletion (GWD). Ruess *et al*.^1^ did this by partitioning U.S. Geological Survey (USGS) irrigation data^2^ through the use of PCR-GLOBWB 2^3^ with local inputs. Since USGS irrigation data is partitioned, this means that IWU represents a withdrawal use of water for irrigation, since the USGS water use database is in terms of water withdrawals. This makes IWU values different to many other estimates of water use in the literature, since they are a withdrawal rather than a consumptive water use, such as the water footprints presented by Mekonnen and Hoekstra^4^.

This paper is distinct from the original Ruess *et al*.^1^ publication in one significant way: a different USGS irrigation variable is used. Specifically, this study uses the USGS variable ‘irrigation – total’, while Ruess *et al*.^1^ used the USGS variable ‘irrigation – crop’. There are two major differences in these USGS variables: 1. The ‘irrigation — crop’ variable is defined to be strictly crop irrigation, while ‘irrigation — total’ includes crop irrigation, as well as irrigation for golf courses and parks. 2. The ‘irrigation — crop’ dataset is missing values for some U.S. states, including some significant agricultural states, whereas the ‘irrigation — total’ dataset has full spatial coverage across the country^2^. The 14 states that are not included in the USGS ‘irrigation — crop’ dataset, and thus are not in Ruess *et al*.^1^, are: Arkansas, Hawaii, Louisiana, Mississippi, Missouri, Montana, Nebraska, New Jersey, North Dakota, Oklahoma, South Dakota, Texas, Wisconsin, and Wyoming.

The original study by^1^ selected the crop irrigation dataset in keeping with the structure of the PCR-GLOBWB 2 model, which quantifies strictly crop-related irrigation (not including irrigation for golf courses or parks)^3^. However, the missing irrigation values for some states in the crop irrigation dataset warrants consideration of the total irrigation dataset, with full spatial coverage across the entire Conterminous United States (CONUS). Some U.S. States may not partition their irrigation water use data into the appropriate crop and total categories, which may explain why they are not included in the USGS crop irrigation dataset. Consequently, the goal of this study is to present a new dataset that has been constructed based on the USGS ‘irrigation — total’ variable across all states instead of the ‘irrigation — crop’ variable as used in the original Ruess *et al*.^1^ publication.

We use ‘irrigation — total’ to obtain new and distinct values for all counties in the CONUS. We anticipate that the findings will largely be comparable to those in^1^, although there may be some important differences. The overarching question we answer in this report is: How does irrigation by crop compare when the USGS variable ‘irrigation — total’ is used instead of ‘irrigation — crop’? The specific questions that we address in this paper are: 1. How do national values of irrigation by crop change when the USGS variable ‘irrigation — total’ is used instead of ‘irrigation — crop’?, 2. How do spatial patterns of irrigation by crop differ when the USGS variable ‘irrigation — total’ is used instead of ‘irrigation — crop’?, and 3. How does irrigation by crop change with time when the USGS variable ‘irrigation — total’ is used instead of ‘irrigation — crop’? IWU values used to address these questions are published in a supporting dataset with this paper.

## Methods

We use an established global hydrological model, PCR-GLOBWB 2^3^, to estimate irrigation water use by crop throughout the United States. We force PCR-GLOBWB 2 with high-resolution data inputs for the CONUS in order to better capture specific crop locations and water demands. Modelled estimates of total irrigation withdrawals (by surface and groundwater sources) are constrained to match the USGS Water Use Database^5^ to ensure our values match this widely used dataset. This means that our estimates of irrigation represent a withdrawal use of water rather than a consumptive use. Note that the USGS dataset provides total irrigation withdrawals that our values are scaled to match, but does not provide irrigation by crop; that is the novelty of our study. The methods presented here are the same as those developed in Ruess *et al*.^1^, but we now scale to ‘irrigation — total’ rather than ‘irrigation — crop’ from the USGS dataset. Please refer to Ruess *et al*.^1^ for additional details.

### Estimating irrigation by crop

**Fig. 1:**
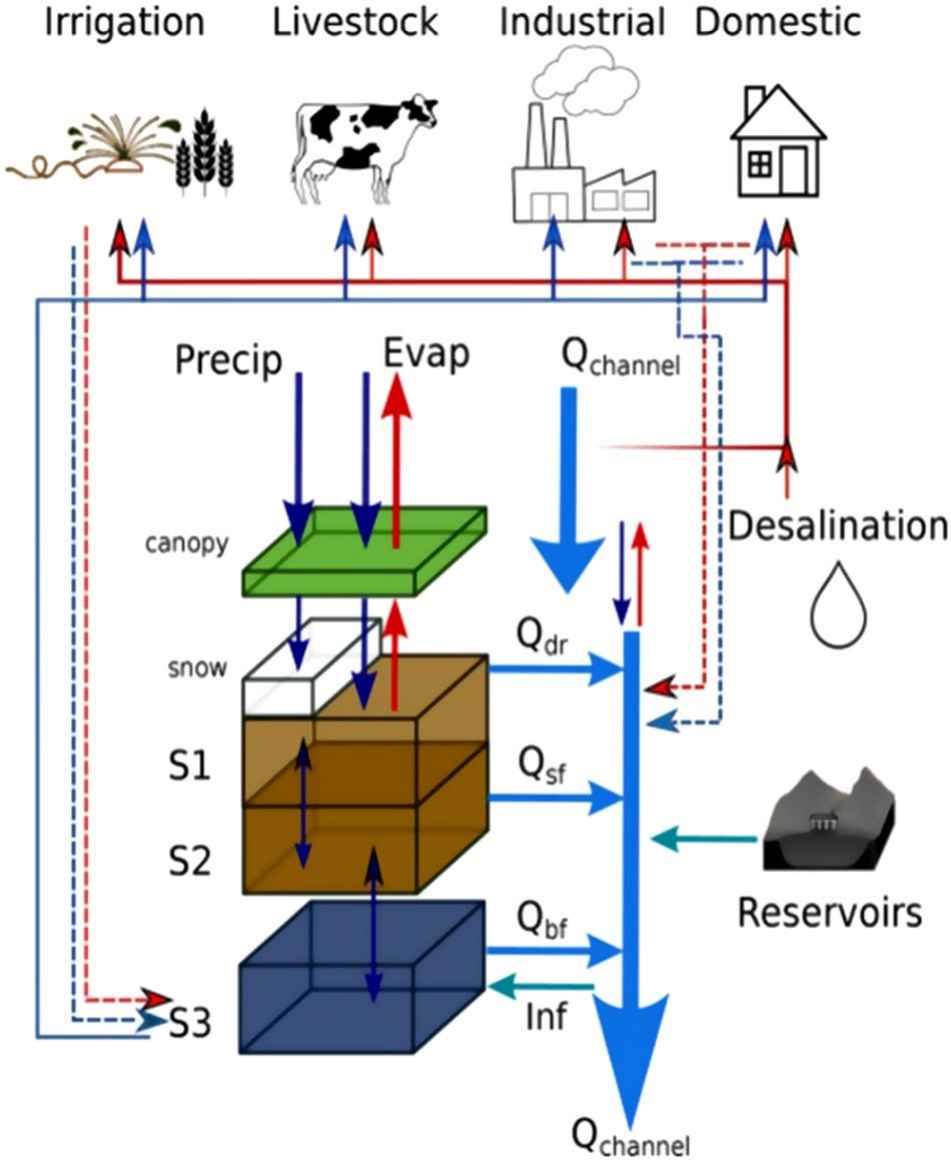
Overview of PCR-GLOBWB 2 modeled states and fluxes for a grid cell; see Fig. 1 in Sutanudjaja *et al*.^3^ for original image. S1, S2 (soil moisture storage), S3 groundwater storage, Qdr (surface runoff from rainfall and snowmelt), Qsf (interflow or stormflow), Qbf (baseflow or groundwater discharge), and Inf (riverbed infiltration to groundwater). The thin red lines indicate surface water withdrawal, the thin blue lines groundwater withdrawal, the thin red dashed lines return flows from surface water use, and the thin blue dashed lines return flows from groundwater use.

The PCR-GLOBWB 2 model is used to estimate irrigation by crop, water source, and year throughout the United States. PCR-GLOBWB 2 is a state-of-the-art grid-based global hydrology and water resources model. PCR-GLOBWB 2 is run on a 5 arc-min spatial resolution and daily temporal resolution, with hydrodynamic river routing at a sub-daily timestep^3^. PCR-GLOBWB 2 simulates moisture storage in two vertically stacked soil layers (S1 and S2 in Fig. 1), as well as the water exchange among the soil, atmosphere, and underlying groundwater reservoir (S3 in Fig. 1). In addition to irrigation, PCR-GLOBWB 2 also considers water use for livestock, industry, and households sectors. See Fig. 1 for a schematic of the PCR-GLOBWB 2 modelled states and fluxes for a single grid-cell. Full details of PCR-GLOBWB 2 are provided in^3^.

Irrigation water is partitioned into Surface Water Withdrawals (SWW) and Groundwater Withdrawals (GWW). GWW is modeled as overall changes in groundwater storage dynamics, with GWW occurring only when surface water is insufficient to meet crop water demands. Groundwater Depletion (GWD) is calculated as GWW minus modeled groundwater recharge, where recharge is modeled as percolation minus capillary rise, accounting for region-specific aquifer properties and includes concentrated recharge from surface water bodies in case groundwater storage contributes to non-renewable groundwater use^3^.

We estimate irrigation water use for all 5 arc-min grid-cells across the CONUS. Irrigation demands for each grid-cell is calculated based on the crops located in that cell, and the evaporative requirements given the climate inputs. Water demands are specific to each crop based on crop curves constructed from crop coefficients and crop calendars (see Ruess *et al*.^1^ for details). The model estimates how much crop water demand is met by precipitation. Remaining demand is then filled from available surface water followed by groundwater stores. Groundwater recharge is finally modeled, enabling us to calculate groundwater depletion (the difference between withdrawals and recharge). Grid-cell level estimates are aggregated to counties using zonal statistics.

Note that we do not make any modifications to the structure of the PCR-GLOBWB 2 model. The novelty of this study is in forcing the model with high-resolution inputs available for the CONUS to better represent local irrigation demands. The input data are detailed in Table 1. We also expand the resolution of crops. This means that we expand from the original 2 crop categories (i.e., non-paddy and paddy crops) estimated in^3^, to 20 crops with additional information on crop locations, crop coefficients, and crop calendars. We thus run the PCR-GLOBWB 2 model with CONUS-specific agricultural and climate forcing data to estimate crop-specific IWU from both surface water and groundwater. For additional modeling details and validation see Ruess *et al*.^1^.

**Table 1:** High-resolution data on climate and crop characteristics for the Continental United States.

Data	PCR-GLOBWB 2	PCR-CONUS^1^
Crop locations	MIRCA^12^	CropScape^13^
Climate	WFDE5^14^	GridMET^15^
Crop calendars	MIRCA^12^	CCD^16^
Crop coefficients	MIRCA^12^	FAO^17^
Irrigated areas	MIRCA^12^	USGS^5^
Irrigation efficiency	Wada *et al*.^18^	Efficiency^19^

### Constraining modelled estimates with a water use dataset

Estimates of total irrigation water use are constrained to match USGS irrigation data. Specifically, the total IWU for SWW and GWW for each county are scaled to match ‘irrigation — total’ provided by the USGS. This is where this publication differs with Ruess *et al*.^1^: we use ‘irrigation — total’ whereas Ruess *et al*.^1^ used ‘irrigation — crop’. The data dictionary that is provided with the USGS water use dataset is provided as Supplementary Information. Specifically, in Ruess *et al*.^1^ the variables “IC-WGWFr” for “Irrigation-Crop, groundwater withdrawals, fresh, in Mgal/d” and “IC-WSWFr” for “Irrigation-Crop, surface-water withdrawals, fresh, in Mgal/d” were used (see the variables highlighted pink in the Supplementary Information). Now, we use: “IR-WGWFr” for “Irrigation, groundwater withdrawals, fresh, in Mgal/d” and “IR-WSWFr” for “Irrigation, surface-water withdrawals, fresh, in Mgal/d” (the variables highlighted blue in the Supplementary Information). Differences between IC-WGWFr and IR-WGWFR for groundwater irrigation and IC-WSWFR and IR-WSWFr for surface water irrigation drive differences between the two irrigation datasets.

The crop-specific water demands from Section 2.1 are used to partition USGS irrigation withdrawals by crop in the years with USGS data. USGS irrigation data are not by crop, so this study adds this dimension to the literature. The USGS water use dataset is available in 2010 and 2015, so we constrain our model estimates to be identical in these years. This approach ensure that our SWW and GWW estimates sum to credible and widely used USGS irrigation data.

In order to estimate irrigation for years without USGS data, we use a scaling factor approach. We define the scaling factor to be the PCR-GLOBWB 2 model estimates in each year divided by the PCR-GLOBWB 2 model estimates in the reference year. The reference year is 2010 for years 2008–2014 and 2015 for years 2015–2020. Annual values are then established as the USGS value in the reference year multiplied by the scaling factor. We then apply crop-specific fractions from the PCR-GLOBWB 2 model to these scaled annual estimates to estimate SWW and GWW for each county, crop and year. The USGS dataset does not include information on groundwater depletion. We calculate the ratio between modeled estimates of GWD and GWW, and then multiplied this ratio by our scaled GWW values to get GWD estimates. To ensure that the inter-annual growth rate is reasonable, we constrained the scaling factor to be between 0.5 and 2. Please see Ruess *et al*.^1^ for additional details.

## Comparison of modeled results: crop vs. total irrigation

Here we compare our findings with the estimates provided in our original publication (Ruess *et al*.^1^). We also address our research questions and provide estimates of SWW, GWW, and GWD by crop within the CONUS, based on scaling to the ‘irrigation — total’ variable provided in the USGS water use dataset.

### How do national values of irrigation by crop change when the USGS variable ‘irrigation — total’ is used instead of ‘irrigation — crop’?

Table 2 presents irrigation water use volumes for the states that are not included in the USGS ‘irrigation — crop’ dataset. Notably, some major groundwater irrigators are captured by ‘irrigation — total’ that are missing in ‘irrigation — crop’. Montana and Wyoming are large surface water irrigators, that were missing in the original study. Importantly, states within the Mississippi Embayment aquifer (e.g., Arkansas, Mississippi) and the High Plains aquifer (e.g., Nebraska, Texas) were also missing from the original study. Inclusion of these states in this companion paper leads to a more complete picture of irrigation water use by crop, particularly in groundwater-fed systems.

**Table 2:** Irrigation water use volumes for the 14 states that are not included in the USGS ‘irrigation — crop’ dataset in 2015.

State	SWW Total	GWW Total	GWD Total
Arkansas	3.16	12.83	1.92
Louisiana	0.46	0.99	0.48
Mississippi	0.18	2.27	0.04
Missouri	0.10	1.79	0.05
Montana	12.98	0.08	0.05
Nebraska	0.93	7.48	1.05
New Jersey	0.05	0.08	0
North Dakota	0.18	0.14	0.10
Oklahoma	0.20	1.09	0.24
South Dakota	0.10	0.19	0.02
Texas	1.40	6.19	2.31
Wisconsin	0.24	0.40	0
Wyoming	10.02	0.74	0.03

Volumes are in km^3^.

Table 3 shows how IWU values in this study compare with Ruess *et al*.^1^. Values in this study are always higher, since we use the USGS ‘irrigation — total’ variable which is always larger than ‘irrigation — crop’ and contains more states. This explains why total IWU values for surface water withdrawals (SWW) (158%), groundwater withdrawals (GWW) (179%), and groundwater depletion (GWD) (122%) are higher than in Ruess *et al*.^1^. The largest difference by crop between the two studies in terms of percentage is for soybeans: SWW (875%), GWW (1726%) and GWD (477%). By volume, the largest difference is Other SCTG 4 (‘Animal feed and products of animal origin’) for SWW (15.69 km^3^), soybeans for GWW (13.26 km^3^), and cotton for GWD (1.01 km^3^).

**Table 3:** Nationwide total irrigation water use by crop and source.

Crop	SWW Crop	SWW Total	SWW % Change	GWW Crop	GWW Total	GWW % Change	GWD Crop	GWD Total	GWD % Change
Barley	2.69	4.97	184.38	1.33	1.36	102.49	0.84	0.86	101.97
Corn	3.69	5.51	149.41	4.08	11.67	286.01	2.32	3.31	142.62
Cotton	0.76	1.04	137.00	1.32	3.91	296.19	0.95	1.96	205.81
Millet	0.05	0.09	169.49	0.07	0.11	175.30	0.03	0.04	123.18
Oats	0.39	0.51	131.24	0.40	0.54	135.98	0.30	0.35	118.76
Other SCTG 2	0.44	0.52	116.42	0.51	0.61	119.11	0.41	0.44	106.19
Other SCTG 3	8.09	8.56	105.89	11.54	11.93	103.37	8.99	9.10	101.21
Other SCTG 4	25.81	41.50	160.74	12.19	14.04	115.22	9.34	9.92	106.23
Peanuts	0.06	0.08	125.32	0.29	0.35	122.76	0.02	0.04	210.07
Potatoes	1.53	1.62	105.97	1.05	1.10	104.66	0.57	0.58	101.53
Pulses	1.04	1.64	158.34	0.48	0.62	129.13	0.22	0.24	109.37
Rapeseed	0.04	0.08	191.22	0.02	0.03	139.56	0.00	0.01	150.41
Rice	0.28	1.36	491.85	1.59	4.63	290.53	0.94	3.42	365.52
Rye	0.04	0.05	120.89	0.08	0.14	168.85	0.04	0.06	165.59
Sorghum	0.28	0.88	320.23	1.02	3.05	299.21	0.56	0.84	147.94
Soybeans	0.35	3.06	875.70	0.81	14.07	1726.66	0.06	0.29	477.89
Sugarbeets	0.69	1.09	159.14	0.58	0.61	104.72	0.27	0.28	101.28
Sunflower	0.07	0.16	243.29	0.10	0.13	134.49	0.08	0.08	111.13
Sweet Potatoes	0.01	0.01	141.74	0.01	0.02	237.96	0.00	0.00	100.03
Wheat	6.63	10.97	165.30	6.48	9.96	153.73	3.70	4.42	119.58
Total	52.94	83.70	158.11	43.95	78.88	179.50	29.66	36.24	122.22

Sum of crop-only irrigation, total irrigation, and their percent difference. Values for 2015. Volumes in km^3^.

**Fig. 2:**
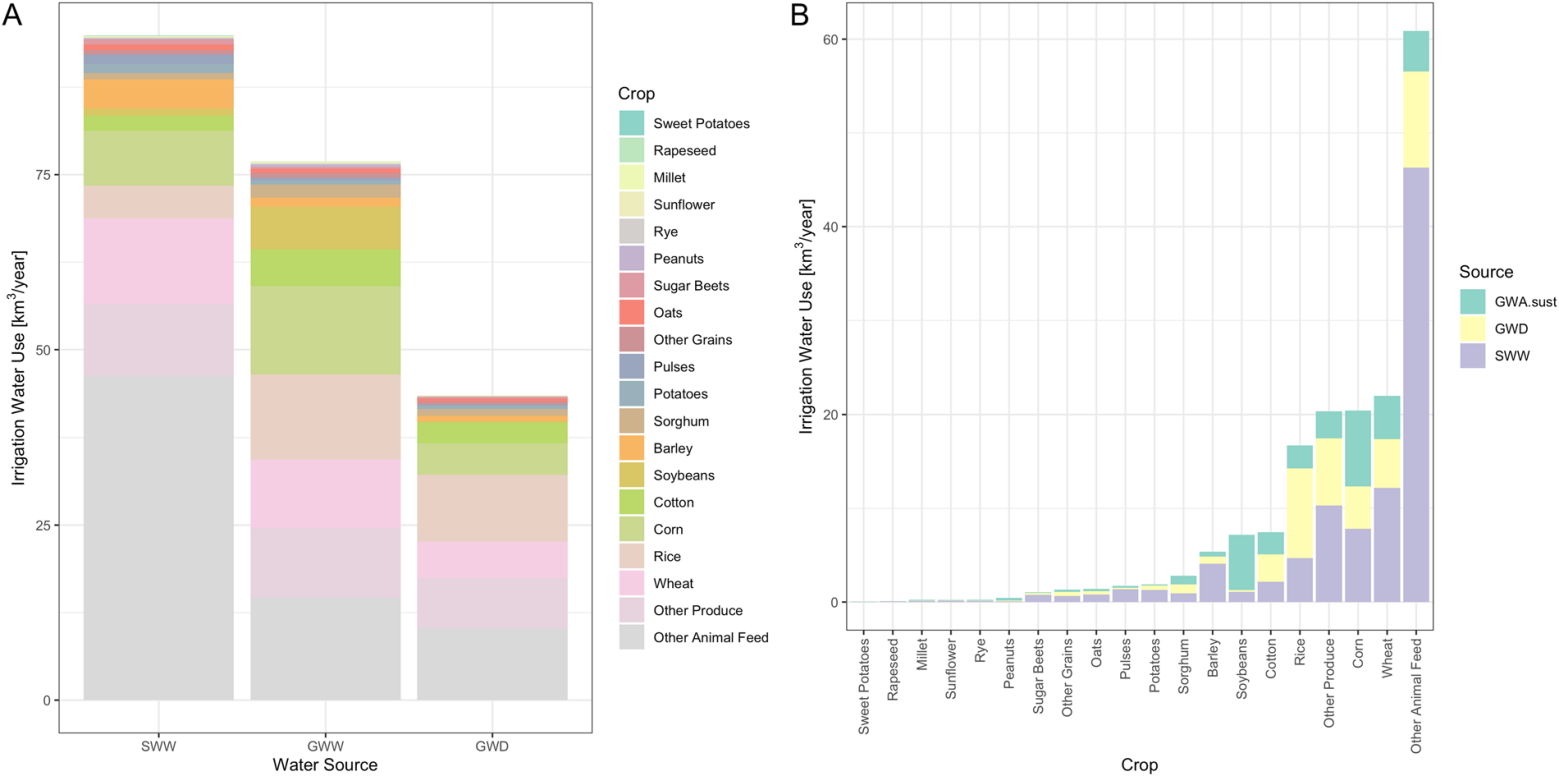
Irrigation Water Use (IWU) [km^3^ yr^−1^] by (**A**) Water Source and (**B**) Crop. IWU is averaged across all study years, 2008–2020. IWU by crop in (**B**) includes a ‘sustainable’ groundwater abstraction variable (GWA_sust), which is the difference between groundwater withdrawals (GWW) and groundwater depletion (GWD).

Figure 2 presents IWU by water source and crop. Figure 2A shows how each water source is broken down by crop. Most IWU comes from SWW, followed by GWW, where approximately half of the latter comes from GWD (note the size of the bars in Fig. 2A). GWW contributes about 80% as much IWU as does SWW (Fig. 1A). Since GWD is a fraction of GWW, we can also see that roughly 55% of all GWW are unsustainably sourced and contribute to GWD, while the remaining 45% is the sustainable portion of total GWW. Figure 2B makes it clear that ‘Other animal feed’ (e.g., hay/alfalfa) is the largest user across all three water sources. ‘Other animal feed’ is responsible for 35% of total IWU, including 49% of SWW, 19% of GWW, and 24% of GWD. The next largest user is wheat (13% total IWU), followed by (in order) corn, ‘other produce’, rice, cotton, soybeans, and barley, with all remaining crops summing to less than 7% of total IWU. Most IWU for ‘other animal feed’ is from SWW, which makes up 75% of its total IWU. There is less variability in GWW between crops, though some crops rely more on unsustainable groundwater, such as rice, ‘other produce’, and ‘other animal feed’; while some crops rely relatively more on sustainable groundwater, such as corn and soybeans.

The rankings of crops in terms of their use of irrigation water is identical to Ruess *et al*.^1^. However, the IWU volumes are larger in this study, since here we look at ‘irrigation — total’ instead of its subset of ‘irrigation — crop’.

### How do spatial patterns of irrigation by crop differ when the USGS variable ‘irrigation — total’ is used instead of ‘irrigation — crop’?

**Fig. 3:**
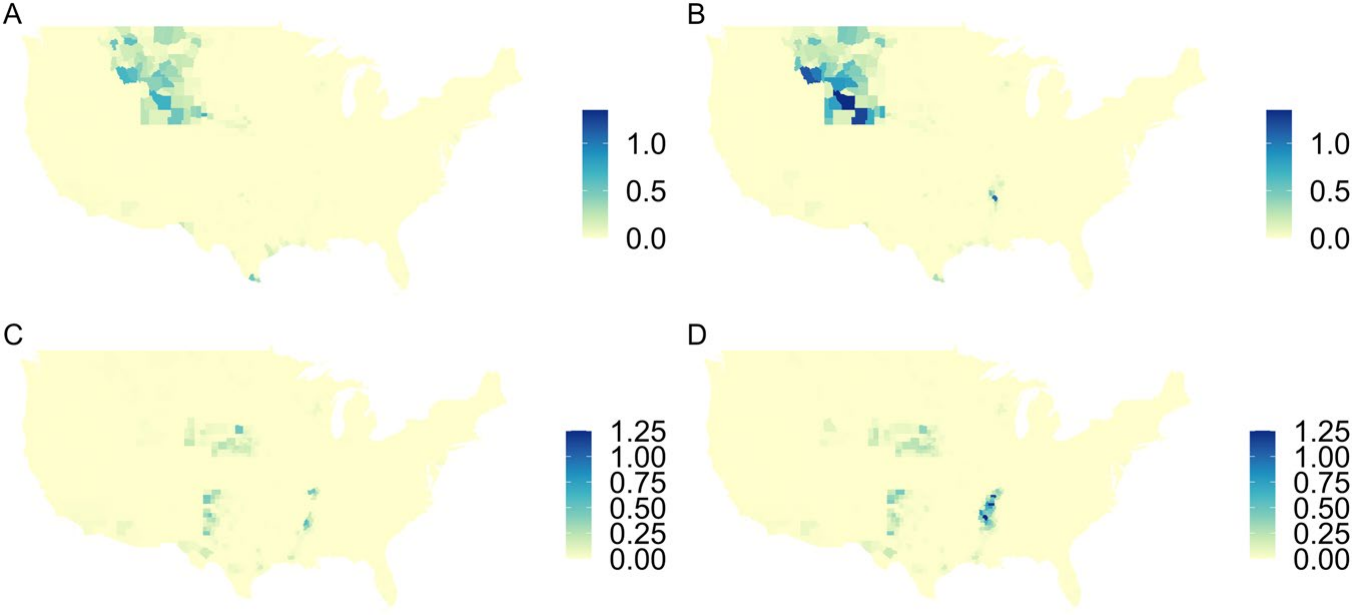
Difference between USGS ‘irrigation — total’ and USGS ‘irrigation — crop’ data [km^3^ yr^−1^]. Maps show ‘irrigation — total’ minus ‘irrigation — crop’ for: (**A**) Surface Water Withdrawals (SWW) in 2010, (**B**) SWW in 2015, (**C**) Groundwater Withdrawals (GWW) in 2010, and (**D**) GWW in 2015.

We map the differences between the two USGS irrigation variables in Fig. 3 to better understand their spatial differences. The difference maps in Fig. 3 are all positive because they show total irrigation minus crop irrigation, and total irrigation is always larger than crop irrigation (since crop irrigation is a subset of total irrigation). Differences are larger in 2015 than in 2010, and are concentrated in the states that do not report crop irrigation data (which are handled as zeros in the crop irrigation map). Importantly, the Mississippi Embayment aquifer has large differences, showing that it is captured in the total irrigation dataset.

**Fig. 4:**
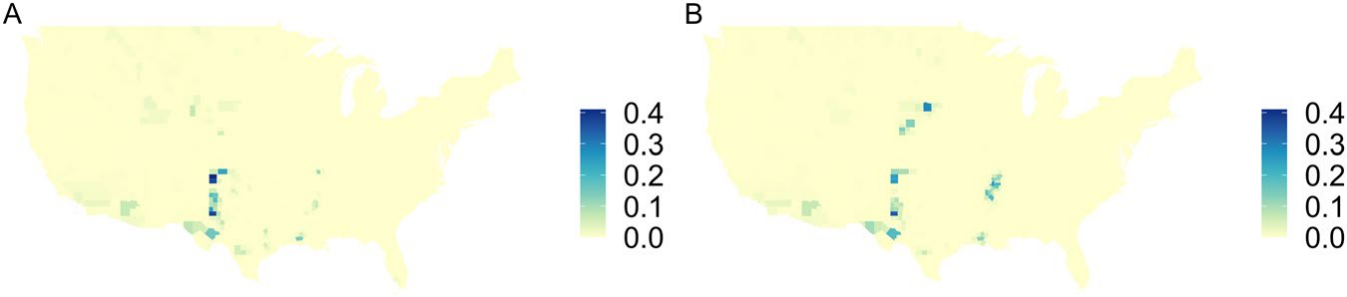
Difference between modeled estimates of Groundwater Depletion (GWD) [km^3^ yr^−1^] in this study and^1^. GWD values from this study minus GWD values from^1^ are shown for: (**A**) 2010 and (**B**) 2015.

The difference in groundwater depletion (GWD) values between Ruess *et al*.^1^ and this study are shown in Fig. 4. We now estimate more GWD because it is a fraction of GWW which increased when total irrigation was used. In particular, there is more GWD throughout the High Plains aquifer, especially in Texas. There is also more GWD in the Mississippi Embayment aquifer. Figure 4 shows that there is more groundwater depletion throughout the country in this study, as compared with Ruess *et al*.^1^, due to increased groundwater irrigation in the total irrigation dataset from USGS.

**Fig. 5:**
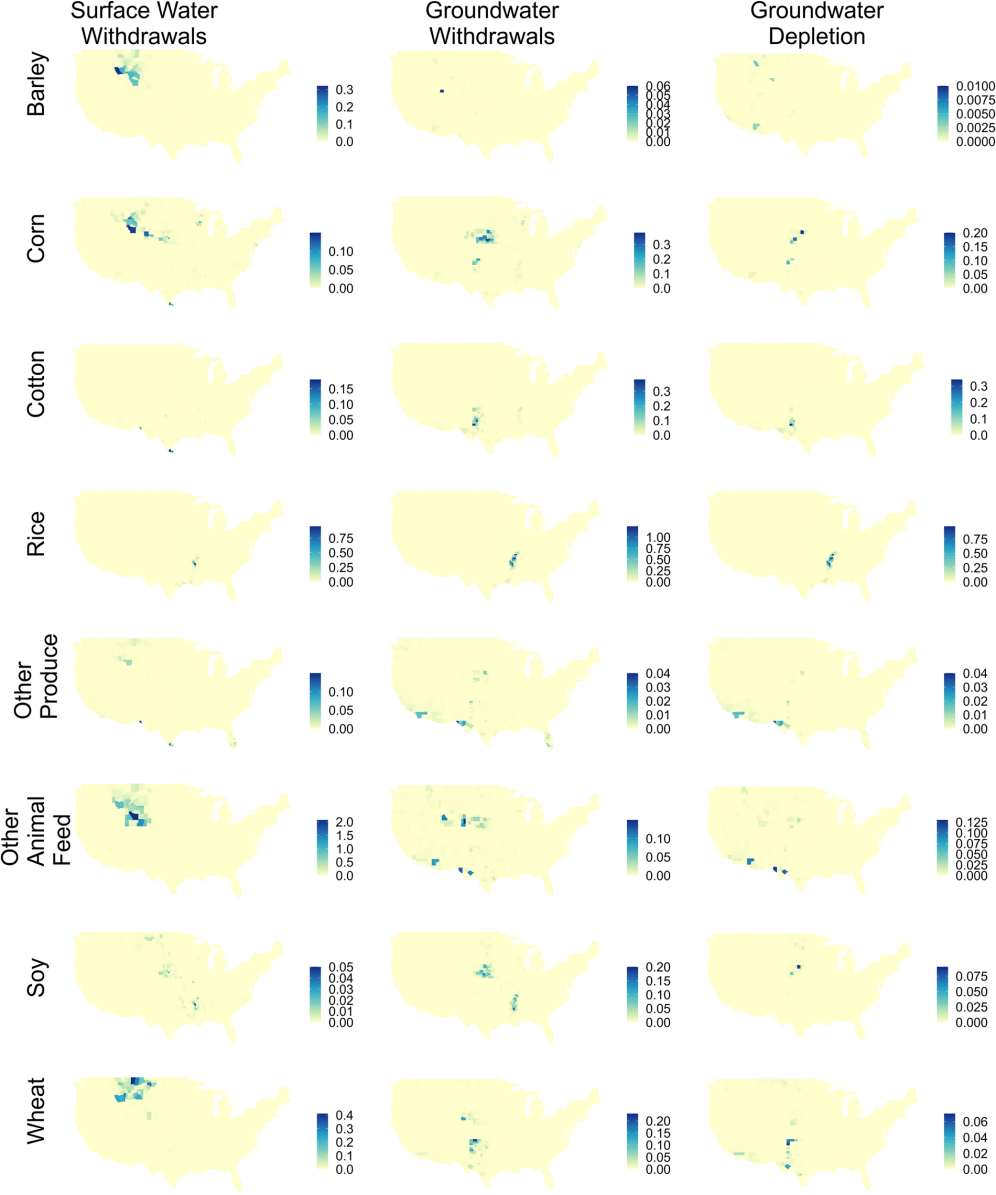
Maps of Irrigation Water Use (IWU) [km^3^ yr^−1^] by water source in 2020. Columns show water source: Surface Water Withdrawals (SWW), Groundwater Withdrawals (GWW), and Groundwater Depletion (GWD). Rows show specific crops: barley, corn, cotton, rice, ‘other produce’, ‘other animal feed’, soy, and wheat.

Irrigation by crop, county, and water source is mapped in Fig. 5 for the crops that use the most irrigation. The spatial distribution of irrigation by crop and source is similar to Ruess *et al*.^1^, though some locations and crops exhibit higher irrigation water use than they did previously. Of particular note is the Mississippi Embayment aquifer, which now exhibits significant irrigation for select crops (rice especially, but soy has significant GWW as well). The southern portion of the High Plains aquifer is also more apparent now, particularly groundwater irrigation of wheat in Texas. This can be explained by the fact that Texas does not report crop irrigation and was thus missing from Ruess *et al*.^1^.

**Fig. 6:**
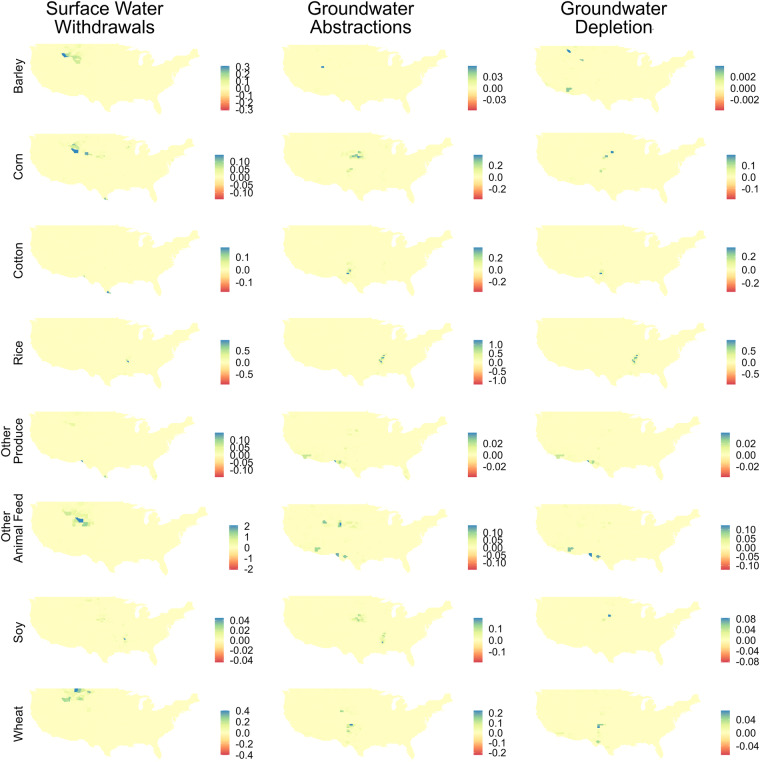
Maps of differences between Irrigation Water Use (IWU) [km^3^ yr^−1^] scaled to ‘irrigation — total’ (this study) vs. ‘irrigation — crop’ (from Ruess *et al*.^1^ for 2020. Columns show water source: Surface Water Withdrawals (SWW), Groundwater Withdrawals (GWW), and Groundwater Depletion (GWD). Rows show crops: barley, corn, cotton, rice, ‘other produce’, ‘other animal feed’, soy, and wheat. Small negative differences exist (largest negative value is -1.5e-3), but note the legends are scaled to be symmetric around zero.

Figure 6 maps differences between between IWU by crop-source from this study compared with Ruess *et al*.^1^. Figure 6 shows that IWU by crop-source from this study are primarily larger than Ruess *et al*.^1^, although there are some small negative values, which means that some of our crop-source estimates are slightly smaller, despite the fact that total IWU values of this study are always larger. This can be attributed to the thresholds used in our inter-annual scaling approach. The largest differences in SWW are around Wyoming, Montana, and Idaho for barley, corn, other animal feed, and wheat. GWW and GWD are instead more scattered depending on the crop in question. Most increases in GWW and GWD around Nebraska are for corn and soy production, for example, while increases in northern Mississippi are for rice and soy. These areas correspond to the High Plains Aquifer and Mississippi Embayment Aquifers, which are key areas of groundwater irrigation that are missing from Ruess *et al*.^1^. Similar comparisons with Ruess *et al*.^1^ are available in the Supplementary Information for all other study years (2008–2020).

### How does irrigation by crop change with time when the USGS variable ‘irrigation — total’ is used instead of ‘irrigation — crop’?

Table 4 presents irrigation water use over time. Total IWU values averaged across the 13-year period are 95 km^3^ (SWW), 77 km^3^ (GWW), and 43 km^3^ (GWD), which are all larger than their counterparts in the original publication. For surface water, the largest volumetric change over time is Other SCTG 04 (‘Other animal feed’) (15.09 km^3^), while it is rapeseed for the largest percent change (236%) with time. Rice has the largest volumetric change for groundwater (9.02 km^3^), but sugarbeets change the most in fractional terms (217%). Again, rice has the biggest volumetric increase in GWD (7 km^3^), with rapeseed increasing the most in percentage terms (433%).

**Table 4:** Irrigation water use by crop and water source over time, from 2008 to 2020.

Crop	Mean SWW	SWW Vol. Change	SWW % Change	Mean GWA	GWA Vol. Change	GWA % Change	Mean GWD	GWD Vol Change	GWD % Change
Barley	4.10	−0.33	−7.87	1.27	0.06	4.87	0.74	−0.14	−19.45
Corn	7.82	−1.95	−20.72	12.63	3.20	27.81	4.52	0.07	1.65
Cotton	2.18	−0.78	−34.86	5.27	1.90	55.88	2.92	0.94	54.27
Millet	0.10	0.04	28.33	0.13	0.05	36.03	0.05	−0.00	−0.21
Oats	0.79	−0.34	−34.24	0.59	−0.10	−14.75	0.38	−0.11	−22.16
Other SCTG 2	0.65	0.20	24.93	0.69	0.56	105.09	0.44	0.26	74.95
Other SCTG 3	10.28	−3.33	−26.66	10.05	2.44	26.17	7.19	3.43	55.79
Other SCTG 4	46.28	15.09	34.45	14.60	4.64	33.22	10.27	2.64	26.28
Peanuts	0.07	0.00	5.67	0.37	−0.06	−13.06	0.08	−0.06	−40.78
Potatoes	1.29	−0.01	−0.56	0.60	0.10	21.44	0.45	0.06	15.47
Pulses	1.35	0.26	23.43	0.36	0.07	25.78	0.17	0.04	34.04
Rapeseed	0.06	0.07	235.98	0.02	0.01	216.90	0.01	0.01	433.47
Rice	4.68	−1.45	−23.01	12.03	9.02	84.29	9.57	6.73	80.82
Rye	0.11	0.02	26.45	0.14	0.11	92.93	0.06	0.02	43.10
Sorghum	0.91	−0.31	−29.44	1.89	−0.40	−16.08	0.99	−0.80	−48.65
Soybeans	1.08	−0.02	−2.78	6.09	0.13	2.55	0.21	0.13	78.17
Sugarbeets	0.76	0.18	30.01	0.26	0.23	216.94	0.19	0.19	203.69
Sunflower	0.14	0.03	26.29	0.11	−0.00	−0.84	0.05	−0.00	−1.73
Sweet Potatoes	0.02	−0.02	−83.45	0.02	0.00	8.11	0.00	−0.00	−97.61
Wheat	12.19	−5.82	−35.69	9.76	−2.06	−18.76	5.18	−2.51	−37.64
Total	94.86	1.52	1.49	76.88	19.93	27.79	43.47	10.92	26.11

Mean irrigation water use over time, volumetric change with time, and percent change with time. Volumes in km^3^.

**Fig. 7:**
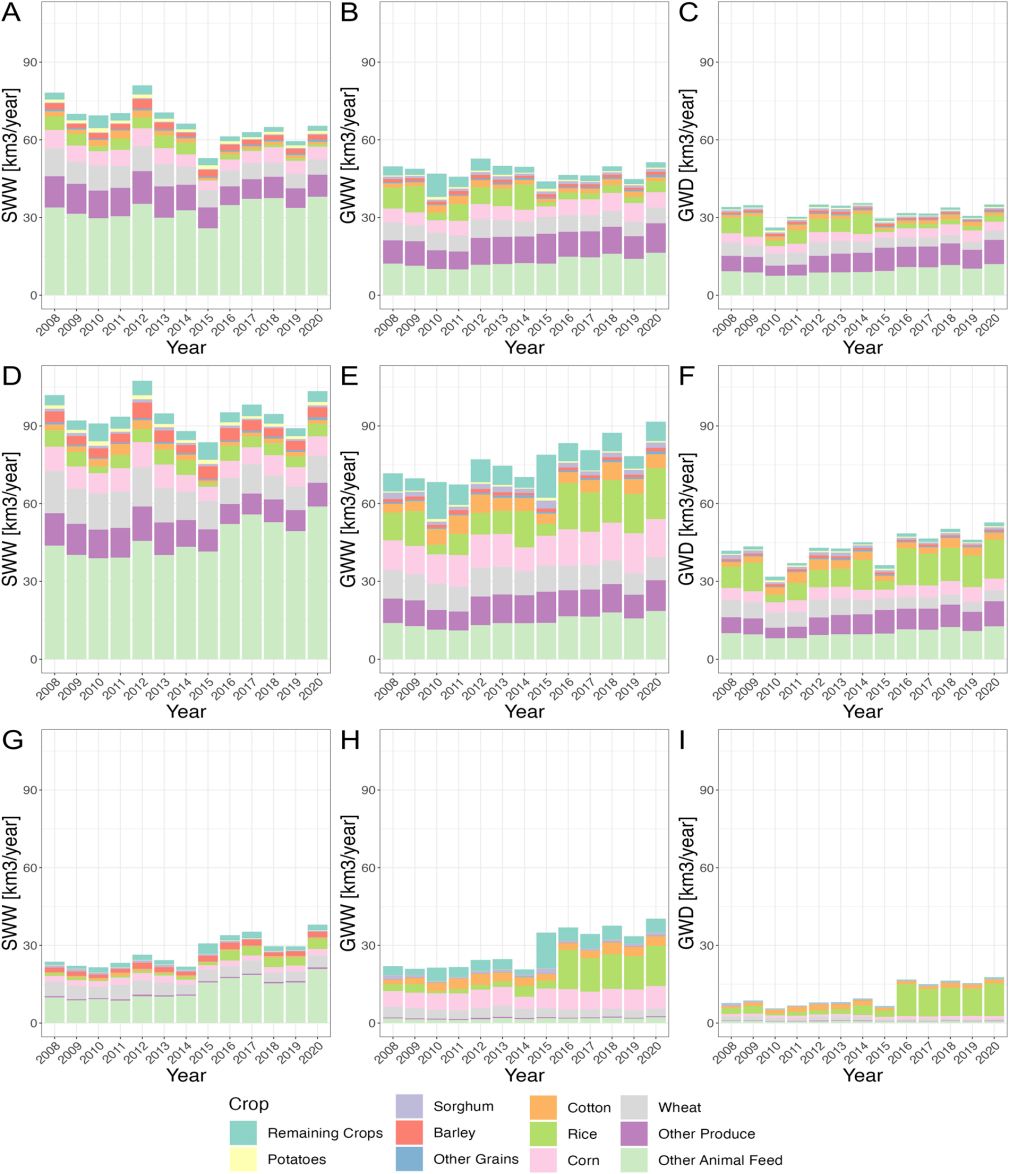
Irrigation Water Use (IWU) by crop and year for Surface Water Withdrawals (SWW), Groundwater Withdrawals (GWW), and Groundwater Depletion (GWD). Row 1 presents prior estimates from^1^, Row 2 shows values from this study, and Row 3 shows the difference between them (Row 2 minus Row 1). Column 1 shows SWW, Column 2 shows GWW, and Column 3 shows GWD.

Figure 7 shows IWU by crop and year over the study time period. Figure 7A shows that total SWW are relatively stable over the study. However, GWW are increasing over time (from ~72 km^3^ yr^−1^ in 2008 to ~92 km^3^ yr^−1^ in 2020) (see Fig. 7E). Similarly, GWD is increasing over time (shown Fig. 7F). Importantly, the change over time in IWU differs from^1^. Now, there is a 1% increase in SWW (compared to a 20% decrease in^1^), a 28% increase in GWW (compared to an 3% increase in^1^), and a 26% increase in GWD (compared to a 3% increase in^1^).

When comparing Fig. 7 to it’s counterpart in^1^ (e.g., Fig. 7), there are a few significant differences. First, the IWU values are larger in this study. This makes sense since total irrigation is now used, instead of crop irrigation. Second, SWW is stable over time in this study (see Fig. 7D), but decreased previously (see Fig. 7A. Notably, GWW and GWD now increase with time (see Fig. 7E,F), while they remainded relatively stable in^1^ (see Fig. 7B,C). Lastly, rice and soy now have much more GWW and GWD due to inclusion of the Mississippi Embayment aquifer, whose states do not report ‘irrigation — crop’.

The bottowm row of Fig. 7G–I shows the differences between the two models over time. The differences are largest for SWW for animal feed, increasing over the study. Differences in GWW and GWD are largest after 2015 for rice. Note that the jump between 2014 and 2015 can partially be explained by the fact that our estimates for 2008–2014 are scaled to USGS data for 2010, which may miss large gains in rice irrigation during this period Ruess *et al*.^1^ applied the same scaling approach, so both models applied the same inter-annual estimation procedure. Changes in rice areas in these years are responsible for the increased irrigation demands.

**Fig. 8:**
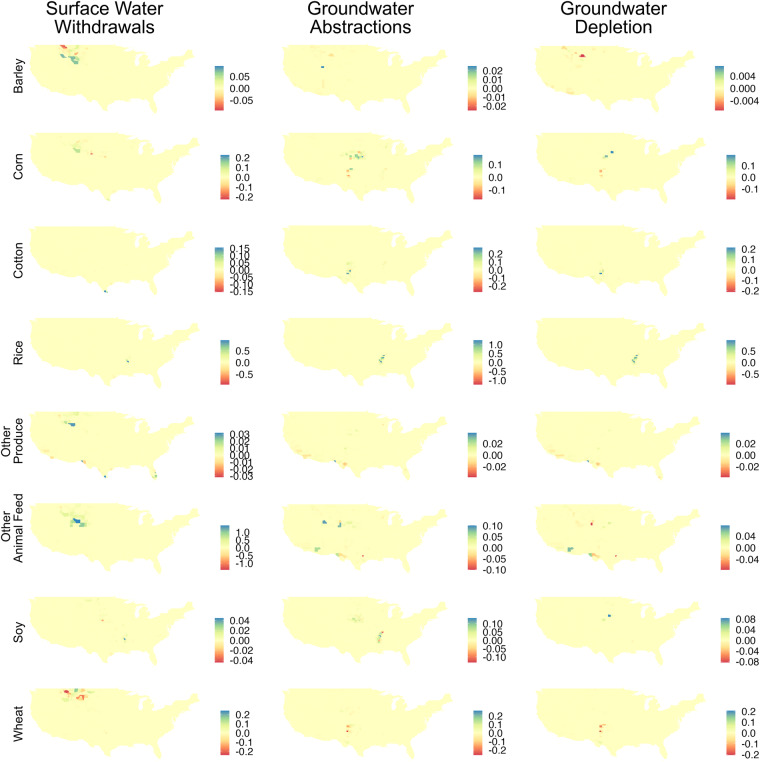
Maps of differences-in-differences between Irrigation Water Use (IWU) [km^3^ yr^−1^] scaled to ‘irrigation — total’ (this study) vs. ‘irrigation — crop’ (from^1^ for 2020–2008. Columns show water source: Surface Water Withdrawals (SWW), Groundwater Withdrawals (GWW), and Groundwater Depletion (GWD). Rows show crops: barley, corn, cotton, rice, ‘other produce’, ‘other animal feed’, soy, and wheat.

To further understand how the models compare over time, we present differences-in-differences maps in Fig. 8. Model differences in 2008 were subtracted from model differences in 2020. Locations with blue shading show areas with increasing differences in IWU in the current study. Counties with red shading indicate that decreasing differences in IWU in the current study. Notably, GWW and GWD differences for rice have increased over the study, concentrated in the Mississippi Embayment. This is different to wheat differences, which have been getting smaller with time.

Reviewing Fig. 8, we see that Other Animal Feed increasingly uses more SWW in this study compared with Ruess *et al*.^1^. There are also large (positive) increases in GWA and GWD for rice, particularly in the Mississippi Embayment, denoting that more groundwater irrigation is being used by rice with time than in Ruess *et al*.^1^. GWW for soybeans is a bit more complex along the Mississippi, with some counties exhibiting positive differences with time, while others have negative differences. Corn GWW difference trends are similarly mixed (both positive and negative depending on the county) in the High Plains aquifer.

## Data Records

The dataset accompanying this study is available at the Illinois Data Bank^6^. The dataset includes Irrigation Water Use (IWU) estimates by crop, county, year, and water source within the Continental United States.

The following files are provided:

- Readme text file. The file ‘total_irrigation_readme.txt’ is provided with information on how to read the data spreadsheets.
- Surface Water Withdrawals spreadsheets. A zipped folder ‘SurfaceWaterWithdrawals.zip’ provides surface water withdrawal information by county, crop, and year.
- Groundwater Withdrawals spreadsheets. A zipped folder ‘GroundwaterWithdrawals.zip’ provides groundwater withdrawal information by county, crop, and year.
- Groundwater Depletion spreadsheets. A zipped folder ‘GroundwaterDepletion.zip’ provides groundwater depletion information by county, crop, and year.

The time period is from 2008 to 2020. Irrigation volumes are given in units of km3. Note that there are three crop categories listed as “other sctg2”, “other sctg3”, and “other sctg4”. These are: Other SCTG 2 = Other Grains; Other SCTG 3 = Other Produce; and Other SCTG 4 = Other Animal Feed.

## Technical Validation

One of the main limitations of our study is that there is no ‘ground-truth’ data to use for validation of our modelled results. Insufficient data for model validation is a common challenge in large-scale hydrologic modeling^7–9^. We instead compare our estimates with other modeled estimates, which is not true validation and has the shortcoming of living in ‘model-land’^10^.

Furthermore, we lean on the comprehensiveness and wide acceptance of the PCR-GLOBWB 2 model in the hydrologic modeling community^3^ as an affirmation that our modeling approach is sound. However, when using a large and complex hydrology model, such as PCR-GLOBWB 2, we recognize that reproducibility is a challenge^11^. This is a general problem that extends beyond the scope of this study. However, in an effort to enhance model transparency and reproducibility the PCR-GLOBWB 2 model has been made open source. The input data has been saved on the PCR-GLOBWB 2 data repository should an external researcher want to build on this study or replicate any of the results.

Similarly, we rely on the widely accepted USGS water use dataset to lend validity to our results. We constrain our values with the USGS water use database of surface water withdrawals and groundwater withdrawals at the county spatial scale. The USGS water use database is only available every 5 years. Consequently, when constraining our estimates to USGS data, we constrained years without USGS data to the closest year with USGS data. This means that we scaled 2010 and 2015 PCR-GLOBWB 2 model estimates directly to their USGS counterparts. But for all other years in our study we scaled PCR-GLOBWB 2 estimates to the nearest available year with USGS data.

Note that some complications arose when scaling PCR-GLOBWB 2 estimates to USGS values. In some instances, the PCR-GLOBWB 2 estimates were much smaller than the USGS values, leading to a very large ‘scaling factor’. We decided to constrain the scaling factor within the range 0.5 to 2 to ensure that we did not force large changes in the data. In other instances, USGS reported positive irrigation but PCR-GLOBWB 2 were zero. To handle this, we calculated a ratio between average PCR-GLOBWB 2 estimates in year of interest to average PCR-GLOBWB 2 estimates in reference year (2010 or 2015). We then applied this ratio to obtain positive irrigation values in those counties. This scaling factor approach enables us to provide estimates that are in line with the USGS database, but also represents an uncertainty that could be improved in future research.

## Usage Notes

We present a dataset of irrigation water use by crop, county, year, and water source for the United States. To do this, we use the framework developed by^1^, but now scale our values to the USGS variable for total irrigation rather than crop irrigation. This means that our irrigation values are generally larger, since crop irrigation is a sub-set of total irrigation. It also improves the spatial coverage of our dataset, since several states do not report crop irrigation to the USGS.

Importantly, several aquifer-dependant locations are now included in our irrigation dataset that are missing in^1^. These include the High Plains aquifer states, Texas and Nebraska, as well as more irrigation reports for the Mississippi Embayment aquifer, with Mississippi and Arkansas included. These changes mean that we now estimate more groundwater withdrawals and groundwater depletion for rice grown in the Mississippi Embayment, and pick up on groundwater irrigation and depletion for wheat production in Texas.

We aim to estimate irrigation by crop, which is why^1^ initially restricted their study to crop irrigation data from the USGS. However, this paper shows that several states only report total irrigation, and that their inclusion is important to accurately capture time trends and the water use of certain crops. Thus, we hope that this companion paper and dataset will be useful to researchers and decision-makers interested in irrigation by crop in the United States.

### Supplementary information

Supporting Information

## Data Availability

PCRaster GLOBal Water Balance model: version 2.0 is a grid-based global hydrology and water resources model developed at Utrecht University and freely available at https://globalhydrology.nl/research/models/pcr-globwb-2-0/.
