# Supplementary material for: Total irrigation by crop in the Continental United States from 2008 to 2020
Source: Sci Data. 2024 Apr 17;11:395. doi: 10.1038/s41597-024-03244-w (PMC11024106; doi:10.1038/s41597-024-03244-w)
Supplement: Supplementary file 1 — Supporting Information [file 41597_2024_3244_MOESM1_ESM.pdf]

Supporting Information for "Total irrigation by crop in the Continental United States from 2008 to 2020"

P.J. Ruess[1], Megan Konar*[1], Niko Wanders[2], and Marc F.P. Bierkens[2,3]

[1]Civil and Environmental Engineering, University of Illinois at Urbana-Champaign

[2]Department of Physical Geography, Utrecht University, Utrecht, The Netherlands

[3]Unit Subsoil and Groundwater Systems, Deltares, Utrecht, The Netherlands

This document contains:

- Table 1: Data dictionary from the U.S. Geological Survey
- Maps of Irrigation Water Use (IWU) differences with Ruess et al. (2023) by year.

*corresponding author

Tables

Table 1: Data dictionary from the U.S. Geological Survey (USGS) water use dataset (USGS, 2018) We define "irrigation – crop" to be the variables "IC-WGWFr" for "Irrigation-Crop, groundwater withdrawals, fresh, in Mgal/d" and "IC-WSWFr" for "Irrigation-Crop, surface-water withdrawals, fresh, in Mgal/d", and which were used in Ruess et al. (2023) (see the variables highlighted pink). We define "irrigation – total" to be the variables "IR-WGWFr" for "Irrigation, groundwater withdrawals, fresh, in Mgal/d" and "IR-WSWFr" for "Irrigation, surface-water withdrawals, fresh, in Mgal/d" which are used in this paper (the variables highlighted blue).

Column Tag	Data Element
STATE	State postal abbreviation
STATEFIPS	State FIPS code
COUNTY	County name
COUNTYFIPS	County FIPS code
FIPS	Concatenated State-county FIPS code
YEAR	Year of data
TP-TotPop	Total population of county, in thousands
PS-GWPop	Public Supply, population served by groundwater, in thousands
PS-SWPop	Public Supply, population served by surface water, in thousands
PS-TOPop	Public Supply, total population served, in thousands
PS-WGWFr	Public Supply, groundwater withdrawals, fresh, in Mgal/d
PS-WGWSa	Public Supply, groundwater withdrawals, saline, in Mgal/d
PS-WGWTo	Public Supply, groundwater withdrawals, total, in Mgal/d
PS-WSWFr	Public Supply, surface-water withdrawals, fresh, in Mgal/d
PS-WSWSa	Public Supply, surface-water withdrawals, saline, in Mgal/d
PS-WSWTo	Public Supply, surface-water withdrawals, total, in Mgal/d
PS-WFrTo	Public Supply, total withdrawals, fresh, in Mgal/d
PS-WSaTo	Public Supply, total withdrawals, saline, in Mgal/d
PS-Wtotl	Public Supply, total withdrawals, total (fresh+saline), in Mgal/d
DO-SSPop	Domestic, self-supplied population, in thousands
DO-WGWFr	Domestic, self-supplied groundwater withdrawals, fresh, in Mgal/d
DO-WSWFr	Domestic, self-supplied surface-water withdrawals, fresh, in Mgal/d
DO-WFrTo	Domestic, total self-supplied withdrawals, fresh, in Mgal/d
DO-SSPCp	Domestic self-supplied per capita use, in gallons/day [DO-WFrTo/DO-SSPop*1000]
DO-PSDel	Domestic, deliveries from Public Supply, in Mgal/d
DO-PSPCp	Domestic, publicly supplied per capita use, in gallons/day [DO-PSDel/PS-TOPop]
DO-TOTAL	Domestic, total use (withdrawals + deliveries)

IN-WGWFr	Industrial, self-supplied groundwater withdrawals, fresh, in Mgal/d
IN-WGWSa	Industrial, self-supplied groundwater withdrawals, saline, in Mgal/d
IN-WGWTo	Industrial, self-supplied groundwater withdrawals, total, in Mgal/d
IN-WSWFr	Industrial, self-supplied surface-water withdrawals, fresh, in Mgal/d
IN-WSWSa	Industrial, self-supplied surface-water withdrawals, saline, in Mgal/d
IN-WSWTo	Industrial, self-supplied surface-water withdrawals, total, in Mgal/d
IN-WFrTo	Industrial, self-supplied total withdrawals, fresh, in Mgal/d
IN-WSaTo	Industrial, self-supplied total withdrawals, saline, in Mgal/d
IN-Wtotl	Industrial, self-supplied total withdrawals, total (fresh+saline), in Mgal/d
IR-WGWFr	Irrigation, groundwater withdrawals, fresh, in Mgal/d
IR-WSWFr	Irrigation, surface-water withdrawals, fresh, in Mgal/d
IR-WFrTo	Irrigation, total withdrawals, fresh, in Mgal/d
IR-IrSpr	Irrigation, acres irrigated, sprinkler, in thousands
IR-IrMic	Irrigation, acres irrigated, microirrigation, in thousands
IR-IrSur	Irrigation, acres irrigated, surface (flood), in thousands
IR-IrTot	Irrigation, acres irrigated, total, in thousands
IC-WGWFr	Irrigation-Crop, groundwater withdrawals, fresh, in Mgal/d
IC-WSWFr	Irrigation-Crop, surface-water withdrawals, fresh, in Mgal/d
IC-WFrTo	Irrigation-Crop, total withdrawals, fresh, in Mgal/d
IC-IrSpr	Irrigation-Crop, acres irrigated, sprinkler, in thousands
IC-IrMic	Irrigation-Crop, acres irrigated, microirrigation, in thousands
IC-IrSur	Irrigation-Crop, acres irrigated, surface (flood), in thousands
IC-IrTot	Irrigation-Crop, acres irrigated, total, in thousands
IG-WGWFr	Irrigation-Golf, groundwater withdrawals, fresh, in Mgal/d
IG-WSWFr	Irrigation-Golf, surface-water withdrawals, fresh, in Mgal/d
IG-WFrTo	Irrigation-Golf, total withdrawals, fresh, in Mgal/d
IG-IrSpr	Irrigation-Golf, acres irrigated, sprinkler, in thousands
IG-IrMic	Irrigation-Golf, acres irrigated, microirrigation, in thousands
IG-IrSur	Irrigation-Golf, acres irrigated, surface (flood), in thousands
IG-IrTot	Irrigation-Golf, acres irrigated, total, in thousands
LI-WGWFr	Livestock, groundwater withdrawals, fresh, in Mgal/d
LI-WSWFr	Livestock, surface-water withdrawals, fresh, in Mgal/d
LI-WFrTo	Livestock, total withdrawals, fresh, in Mgal/d
AQ-WGWFr	Aquaculture, groundwater withdrawals, fresh, in Mgal/d
AQ-WGWSa	Aquaculture, groundwater withdrawals, saline, in Mgal/d
AQ-WGWTo	Aquaculture, groundwater withdrawals, total, in Mgal/d
AQ-WSWFr	Aquaculture, surface-water withdrawals, fresh, in Mgal/d

AQ-WSWSa	Aquaculture, surface-water withdrawals, saline, in Mgal/d
AQ-WSWTo	Aquaculture, surface-water withdrawals, total, in Mgal/d
AQ-WFrTo	Aquaculture, total withdrawals, fresh, in Mgal/d
AQ-WSaTo	Aquaculture, total withdrawals, saline, in Mgal/d
AQ-WTotl	Aquaculture, total withdrawals, total (fresh+saline), in Mgal/d
MI-WGWFr	Mining, groundwater withdrawals, fresh, in Mgal/d
MI-WGWSa	Mining, groundwater withdrawals, saline, in Mgal/d
MI-WGWTo	Mining, groundwater withdrawals, total, in Mgal/d
MI-WSWFr	Mining, surface-water withdrawals, fresh, in Mgal/d
MI-WSWSa	Mining, surface-water withdrawals, saline, in Mgal/d
MI-WSWTo	Mining, surface-water withdrawals, total, in Mgal/d
MI-WFrTo	Mining, total withdrawals, fresh, in Mgal/d
MI-WSaTo	Mining, total withdrawals, saline, in Mgal/d
MI-Wtotl	Mining, total withdrawals, total (fresh+saline), in Mgal/d
PT-WGWFr	Thermoelectric, groundwater withdrawals, fresh, in Mgal/d
PT-WGWSa	Thermoelectric, groundwater withdrawals, saline, in Mgal/d
PT-WGWTo	Thermoelectric, groundwater withdrawals, total, in Mgal/d
PT-WSWFr	Thermoelectric, surface-water withdrawals, fresh, in Mgal/d
PT-WSWSa	Thermoelectric, surface-water withdrawals, saline, in Mgal/d
PT-WSWTo	Thermoelectric, surface-water withdrawals, total, in Mgal/d
PT-WFrTo	Thermoelectric, total withdrawals, fresh, in Mgal/d
PT-WSaTo	Thermoelectric, total withdrawals, saline, in Mgal/d
PT-Wtotl	Thermoelectric, total withdrawals, total (fresh+saline), in Mgal/d
PT-Power	Thermoelectric, power generated, in gigawatt-hours
PO-WGWFr	Thermoelectric once-through, groundwater withdrawals, fresh, in Mgal/d
PO-WGWSa	Thermoelectric once-through, groundwater withdrawals, saline, in Mgal/d
PO-WGWTo	Thermoelectric once-through, groundwater withdrawals, total, in Mgal/d
PO-WSWFr	Thermoelectric once-through, surface-water withdrawals, fresh, in Mgal/d
PO-WSWSa	Thermoelectric once-through, surface-water withdrawals, saline, in Mgal/d
PO-WSWTo	Thermoelectric once-through, surface-water withdrawals, total, in Mgal/d
PO-WFrTo	Thermoelectric once-through, total withdrawals, fresh, in Mgal/d
PO-WSaTo	Thermoelectric once-through, total withdrawals, saline, in Mgal/d
PO-WTotl	Thermoelectric once-through, total withdrawals, total, in Mgal/d
PO-Power	Thermoelectric once-through, power generated, in gigawatt-hours
PC-WGWFr	Thermoelectric recirculation, groundwater withdrawals, fresh, in Mgal/d
PC-WGWSa	Thermoelectric recirculation, groundwater withdrawals, saline, in Mgal/d
PC-WGWTo	Thermoelectric recirculation, groundwater withdrawals, total, in Mgal/d

PC-WSWFr	Thermoelectric recirculation, surface-water withdrawals, fresh, in Mgal/d
PC-WSWSa	Thermoelectric recirculation, surface-water withdrawals, saline, in Mgal/d
PC-WSWTo	Thermoelectric recirculation, surface-water withdrawals, total, in Mgal/d
PC-WFrTo	Thermoelectric recirculation, total withdrawals, fresh, in Mgal/d
PC-WSaTo	Thermoelectric recirculation, total withdrawals, saline, in Mgal/d
PC-WTotl	Thermoelectric recirculation, total withdrawals, total (fresh+saline), in Mgal/d
PC-Power	Thermoelectric recirculation, power generated, in gigawatt-hours
TO-WGWFr	Total groundwater withdrawals, fresh, in Mgal/d
TO-WGWSa	Total groundwater withdrawals, saline, in Mgal/d
TO-WGWTo	Total groundwater withdrawals, total (fresh+saline), in Mgal/d
TO-WSWFr	Total surface-water withdrawals, fresh, in Mgal/d
TO-WSWSa	Total surface-water withdrawals, saline, in Mgal/d
TO-WSWTo	Total surface-water withdrawals, total (fresh+saline), in Mgal/d
TO-WFrTo	Total withdrawals, fresh, in Mgal/d
TO-WSaTo	Total withdrawals, saline, in Mgal/d
TO-WTotl	Total withdrawals, total (fresh+saline), in Mgal/d

1 Figures

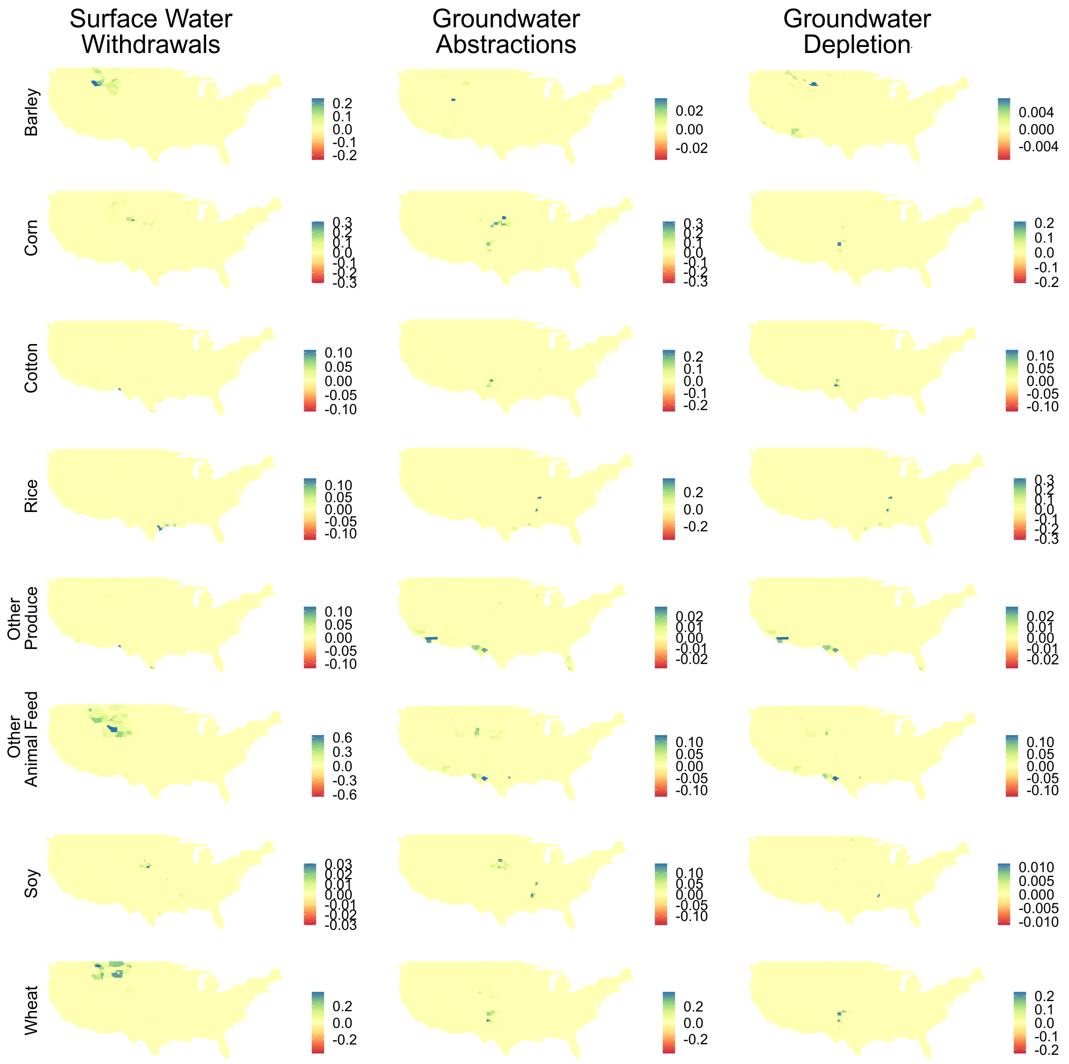

Figure 1: Maps of differences between Irrigation Water Use (IWU) modeled using Total Irrigation (from this work) vs. IWU modeled using Crop Irrigation (from Ruess et al. (2023)), organized by water source in 2008 (km^3 yr^{-1}). Columns show water source: Surface Water Withdrawals (SWW), Groundwater Withdrawals (GWW), and Groundwater Depletion (GWD). Rows show specific crops: barley, corn, cotton, rice, 'other produce', 'other animal feed', soy, and wheat.

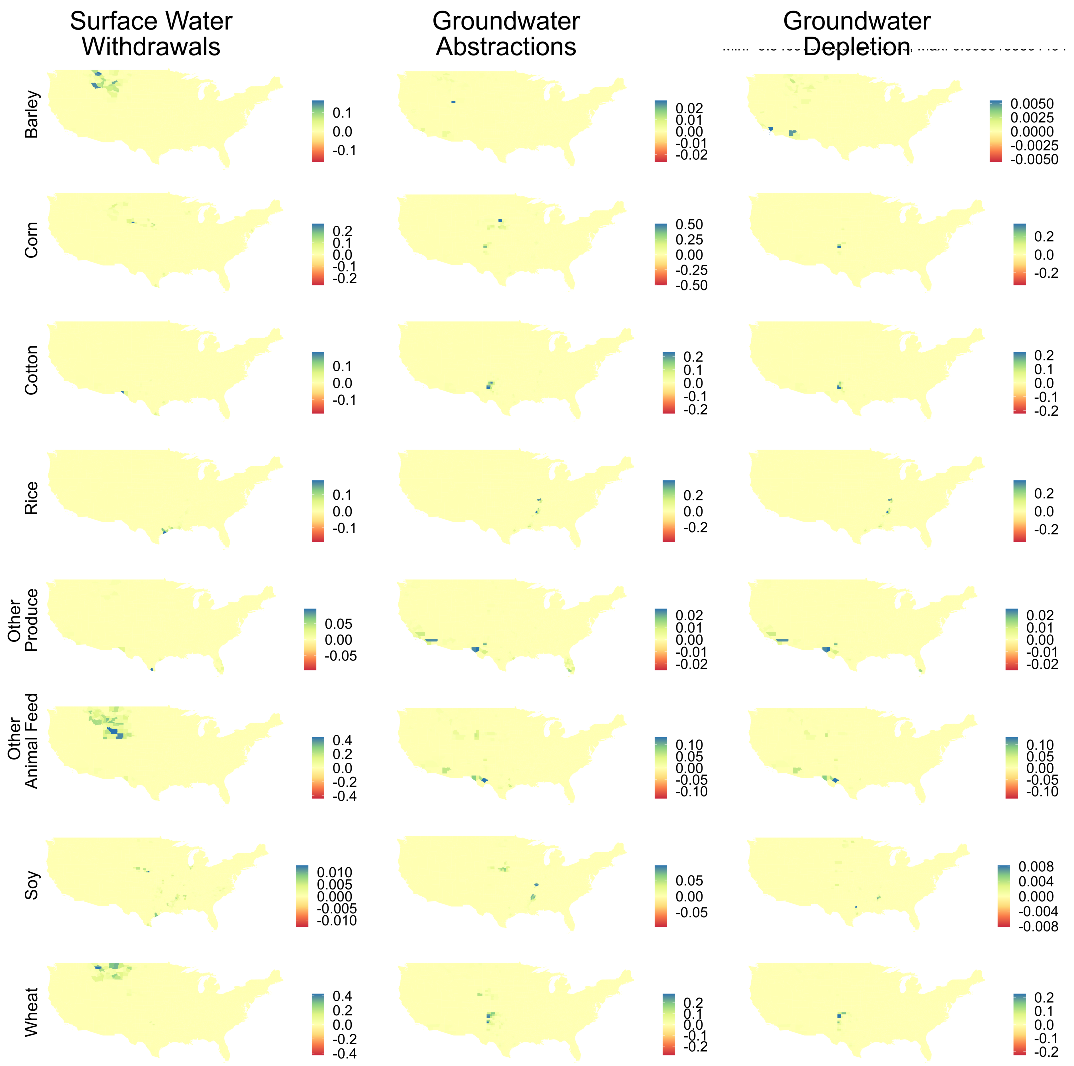

Figure 2: Maps of differences between Irrigation Water Use (IWU) modeled using Total Irrigation (from this work) vs. IWU modeled using Crop Irrigation (from Ruess et al. (2023)), organized by water source in 2009 ($km^3\ yr^{-1}$). Columns show water source: Surface Water Withdrawals (SWW), Groundwater Withdrawals (GWW), and Groundwater Depletion (GWD). Rows show specific crops: barley, corn, cotton, rice, 'other produce', 'other animal feed', soy, and wheat.

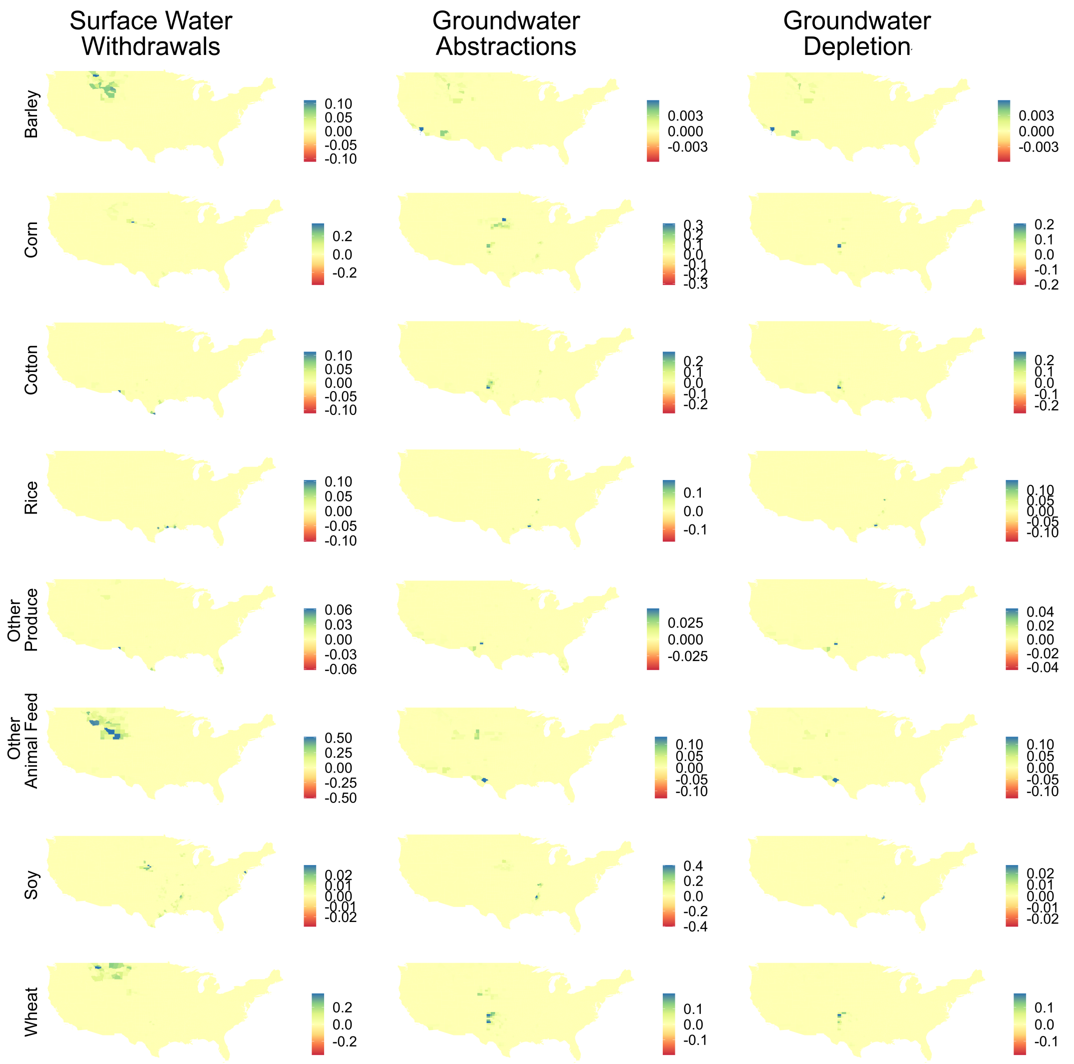

Figure 3: Maps of differences between Irrigation Water Use (IWU) modeled using Total Irrigation (from this work) vs. IWU modeled using Crop Irrigation (from Ruess et al. (2023)), organized by water source in 2010 (km^3 yr^{-1}). Columns show water source: Surface Water Withdrawals (SWW), Groundwater Withdrawals (GWW), and Groundwater Depletion (GWD). Rows show specific crops: barley, corn, cotton, rice, 'other produce', 'other animal feed', soy, and wheat.

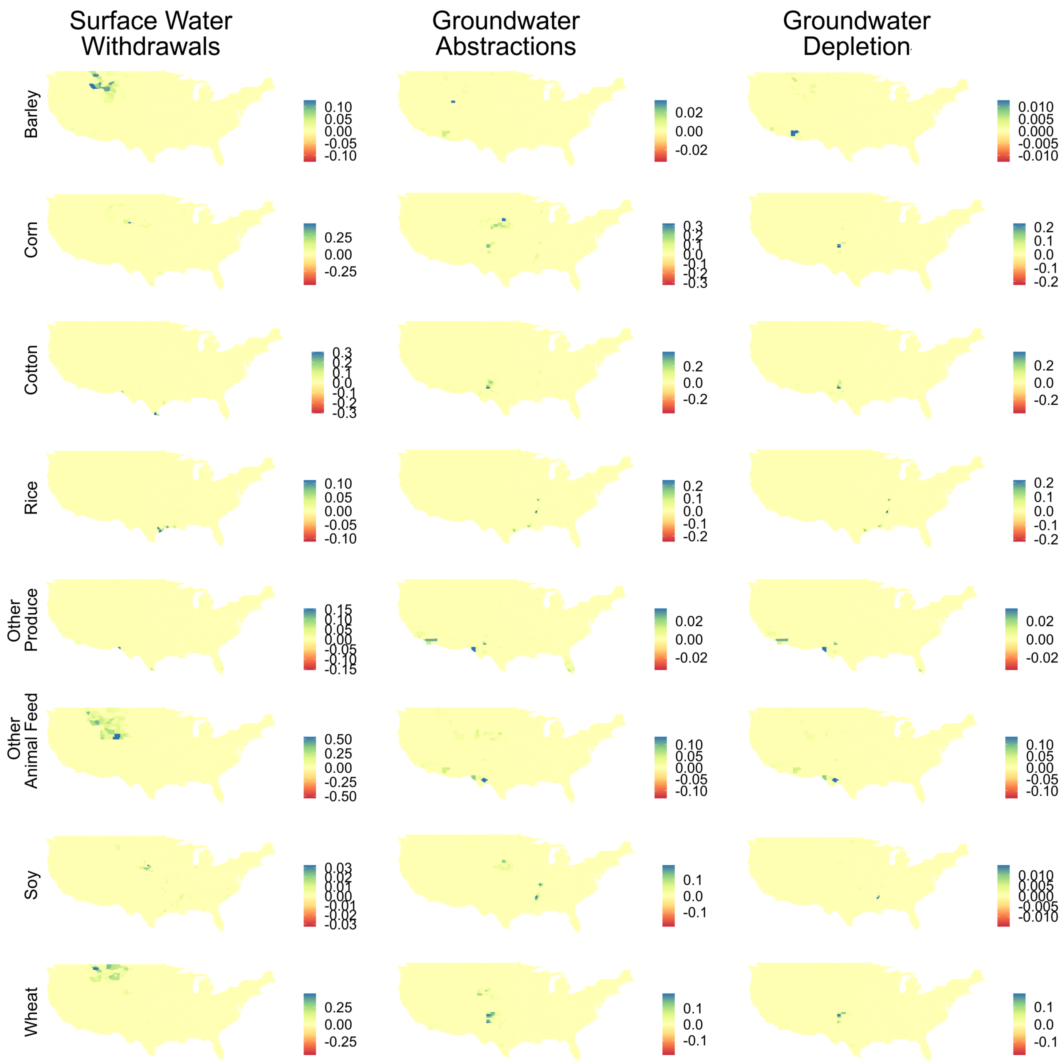

Figure 4: Maps of differences between Irrigation Water Use (IWU) modeled using Total Irrigation (from this work) vs. IWU modeled using Crop Irrigation (from Ruess et al. (2023)), organized by water source in 2011 (km^3 yr^{-1}). Columns show water source: Surface Water Withdrawals (SWW), Groundwater Withdrawals (GWW), and Groundwater Depletion (GWD). Rows show specific crops: barley, corn, cotton, rice, ‘other produce’, ‘other animal feed’, soy, and wheat.

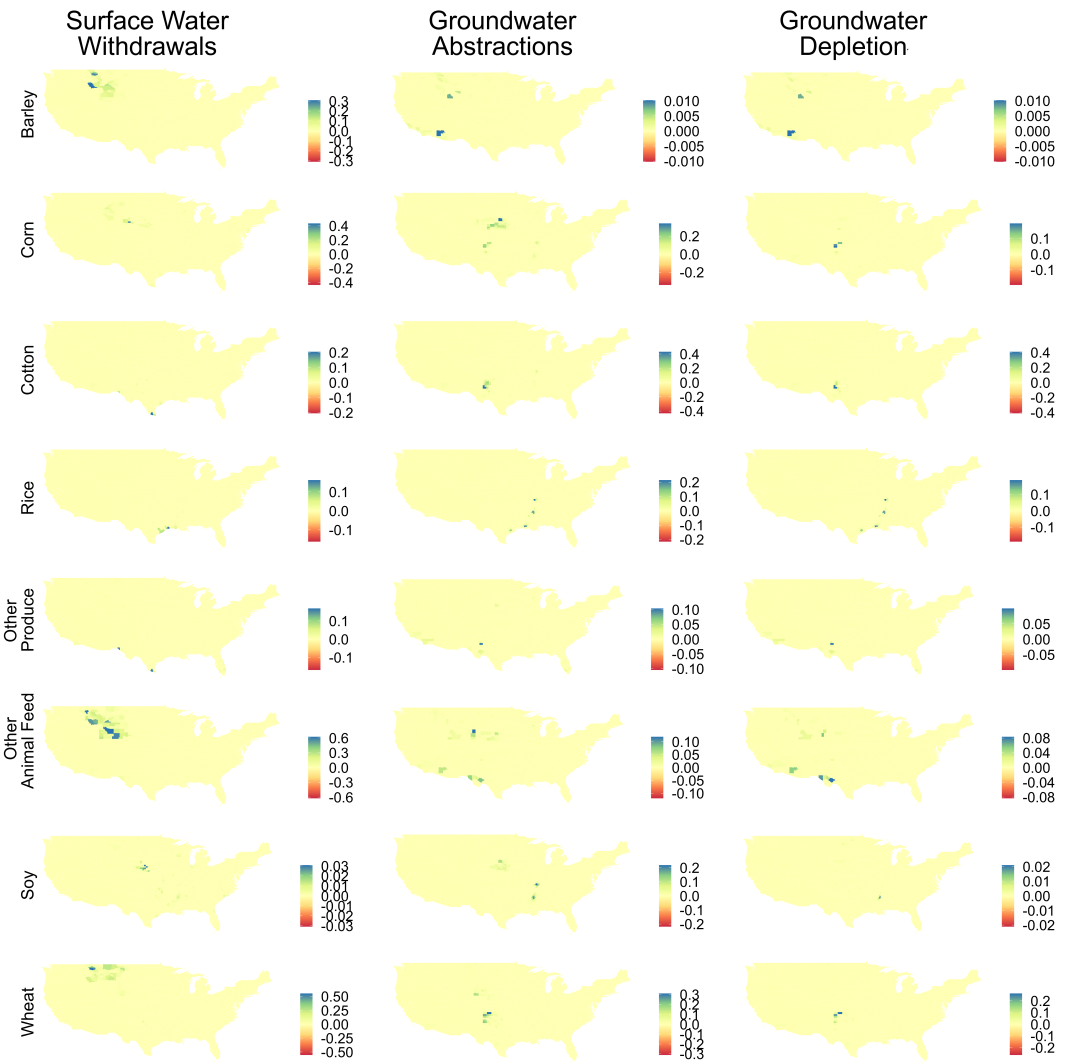

Figure 5: Maps of differences between Irrigation Water Use (IWU) modeled using Total Irrigation (from this work) vs. IWU modeled using Crop Irrigation (from Ruess et al. (2023)), organized by water source in 2012 (km^3 yr^{-1}). Columns show water source: Surface Water Withdrawals (SWW), Groundwater Withdrawals (GWW), and Groundwater Depletion (GWD). Rows show specific crops: barley, corn, cotton, rice, 'other produce', 'other animal feed', soy, and wheat.

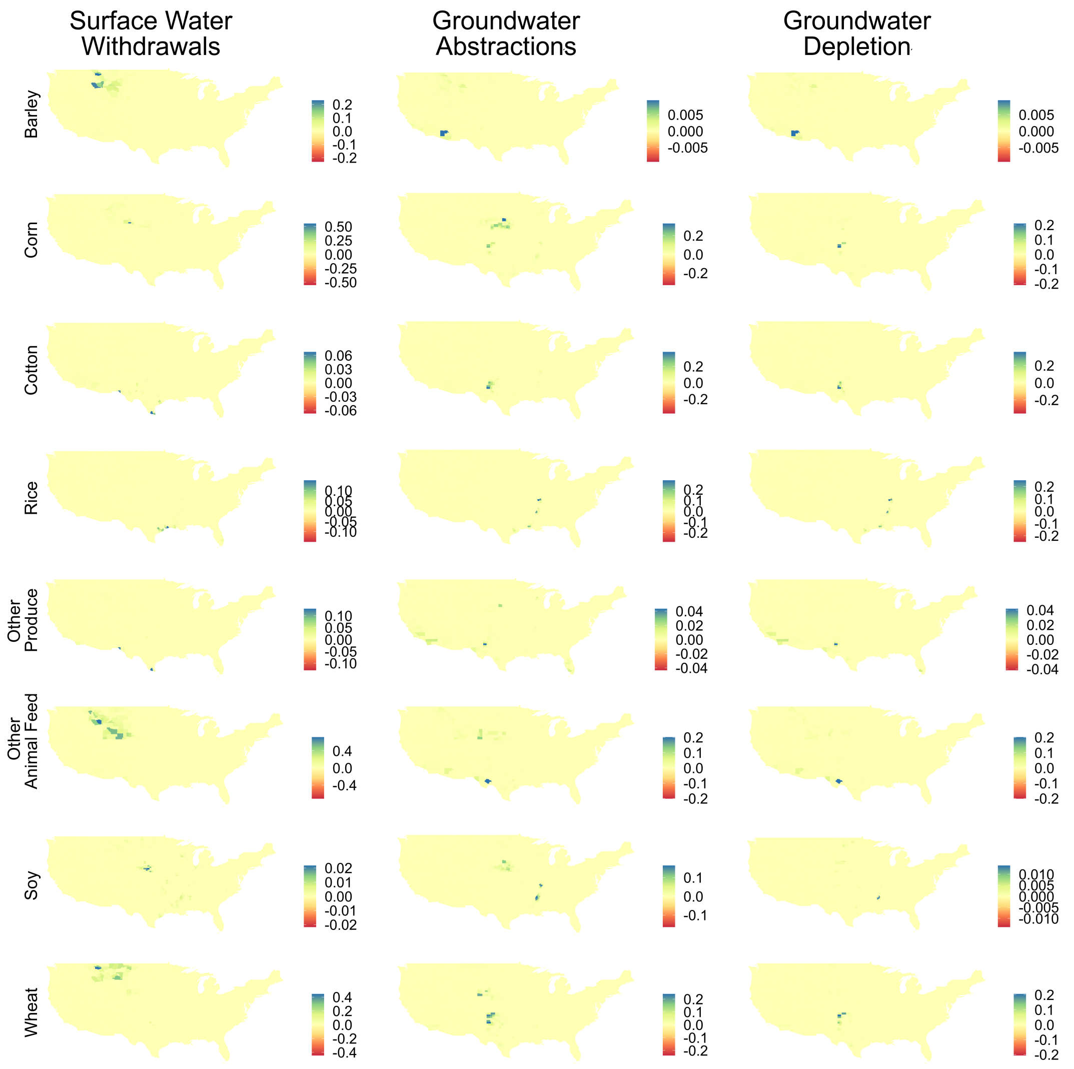

Figure 6: Maps of differences between Irrigation Water Use (IWU) modeled using Total Irrigation (from this work) vs. IWU modeled using Crop Irrigation (from Ruess et al. (2023)), organized by water source in 2013 (km^3 yr^{-1}). Columns show water source: Surface Water Withdrawals (SWW), Groundwater Withdrawals (GWW), and Groundwater Depletion (GWD). Rows show specific crops: barley, corn, cotton, rice, 'other produce', 'other animal feed', soy, and wheat.

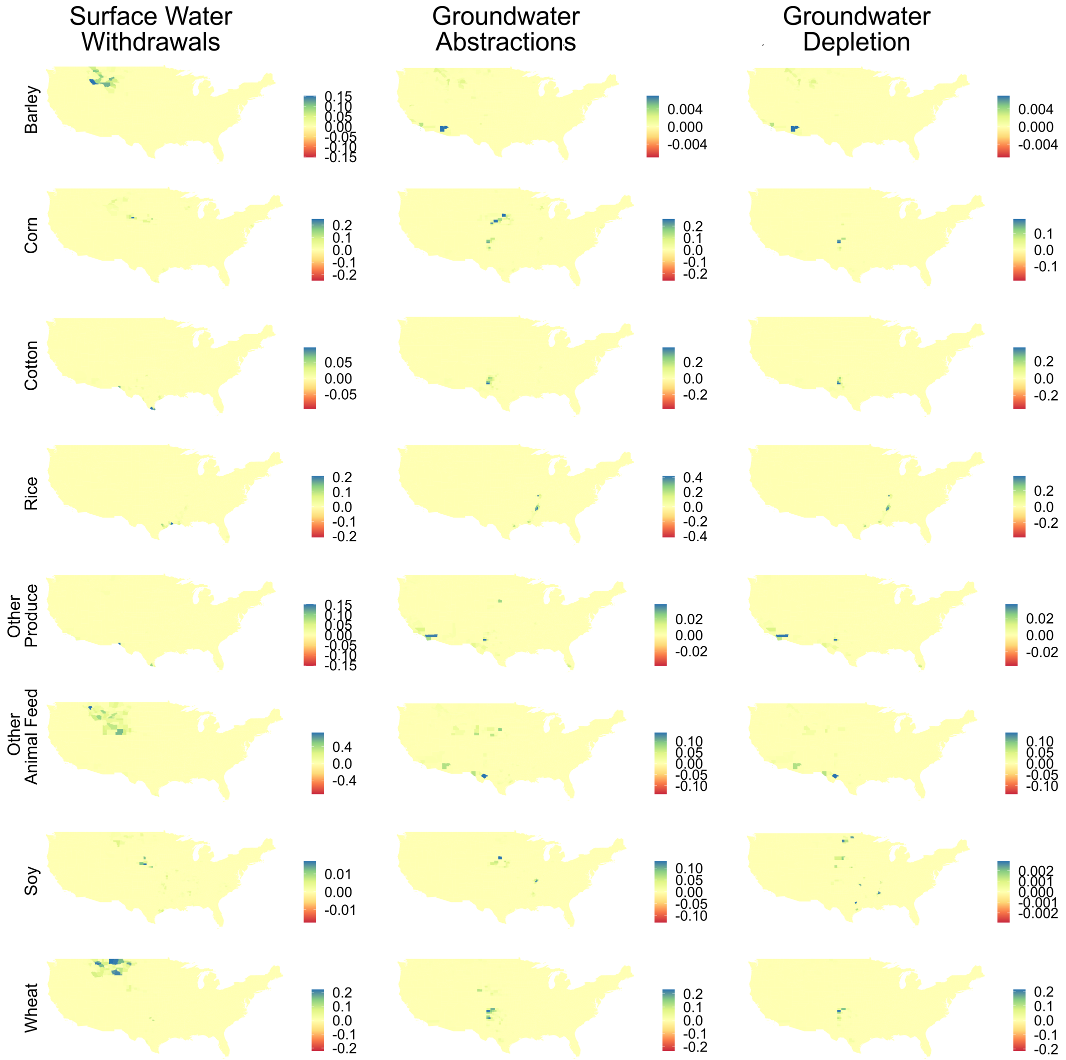

Figure 7: Maps of differences between Irrigation Water Use (IWU) modeled using Total Irrigation (from this work) vs. IWU modeled using Crop Irrigation (from Ruess et al. (2023)), organized by water source in 2014 (km^3 yr^{-1}). Columns show water source: Surface Water Withdrawals (SWW), Groundwater Withdrawals (GWW), and Groundwater Depletion (GWD). Rows show specific crops: barley, corn, cotton, rice, 'other produce', 'other animal feed', soy, and wheat.

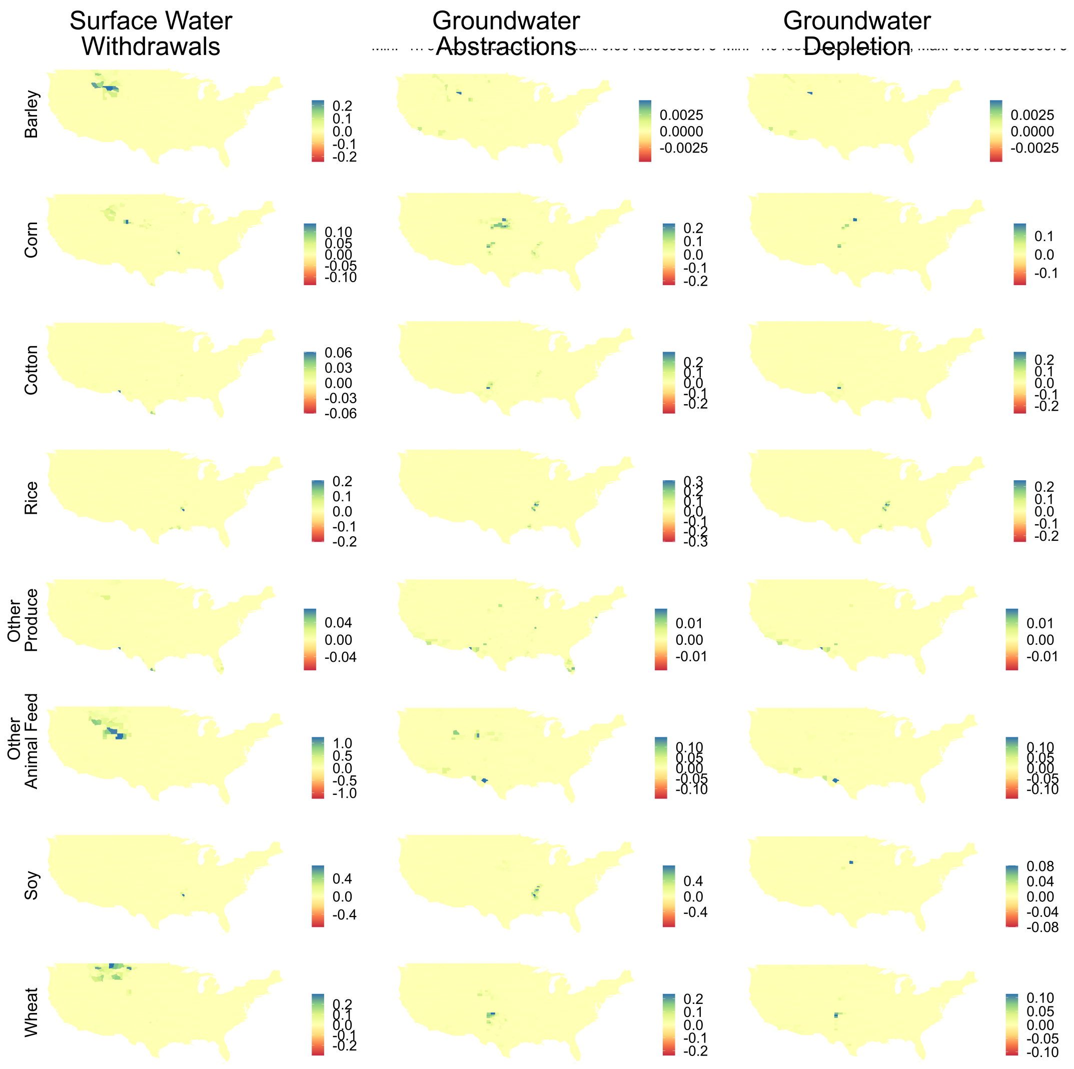

Figure 8: Maps of differences between Irrigation Water Use (IWU) modeled using Total Irrigation (from this work) vs. IWU modeled using Crop Irrigation (from Ruess et al. (2023)), organized by water source in 2015 (km^3 yr^{-1}). Columns show water source: Surface Water Withdrawals (SWW), Groundwater Withdrawals (GWW), and Groundwater Depletion (GWD). Rows show specific crops: barley, corn, cotton, rice, 'other produce', 'other animal feed', soy, and wheat.

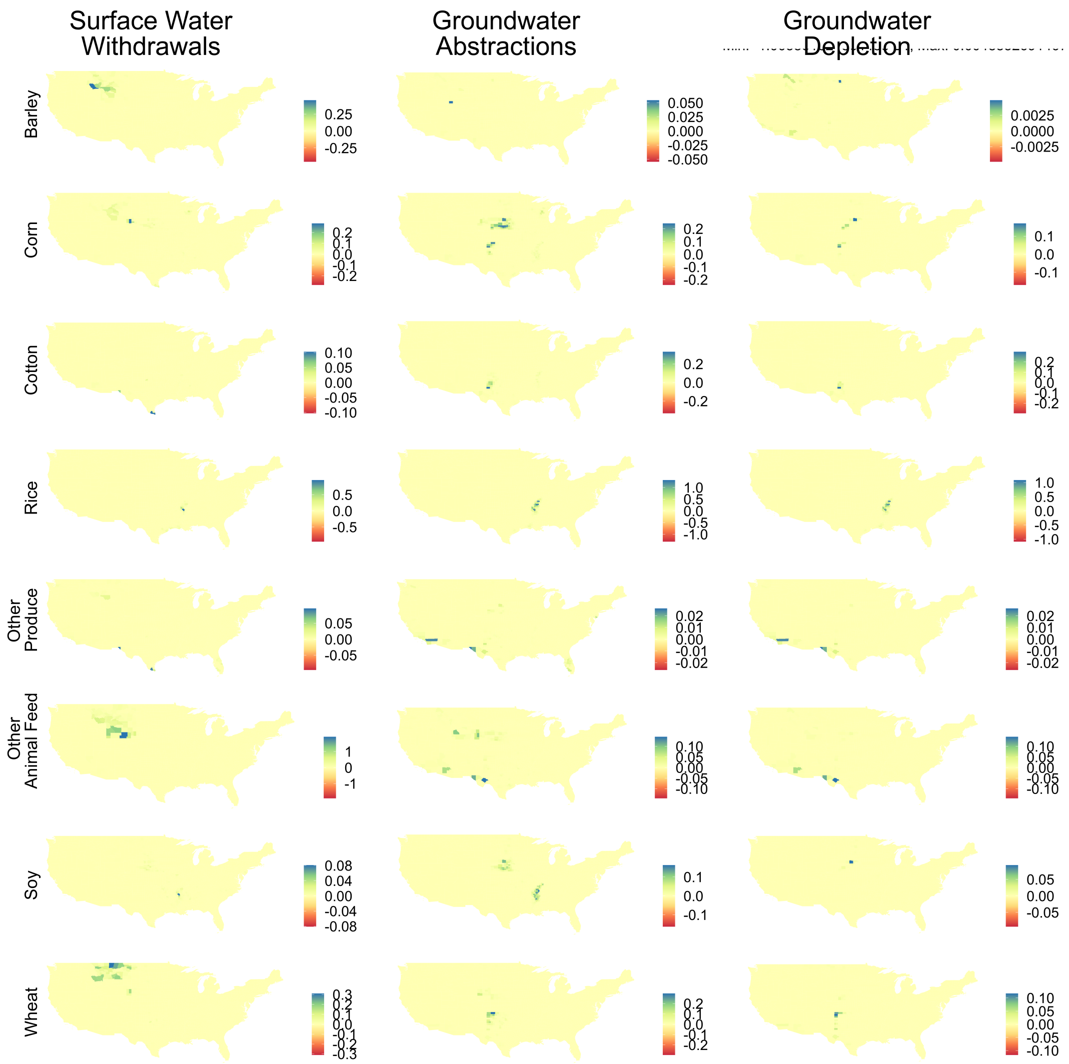

Figure 9: Maps of differences between Irrigation Water Use (IWU) modeled using Total Irrigation (from this work) vs. IWU modeled using Crop Irrigation (from Ruess et al. (2023)), organized by water source in 2016 (km^3 yr^{-1}). Columns show water source: Surface Water Withdrawals (SWW), Groundwater Withdrawals (GWW), and Groundwater Depletion (GWD). Rows show specific crops: barley, corn, cotton, rice, 'other produce', 'other animal feed', soy, and wheat.

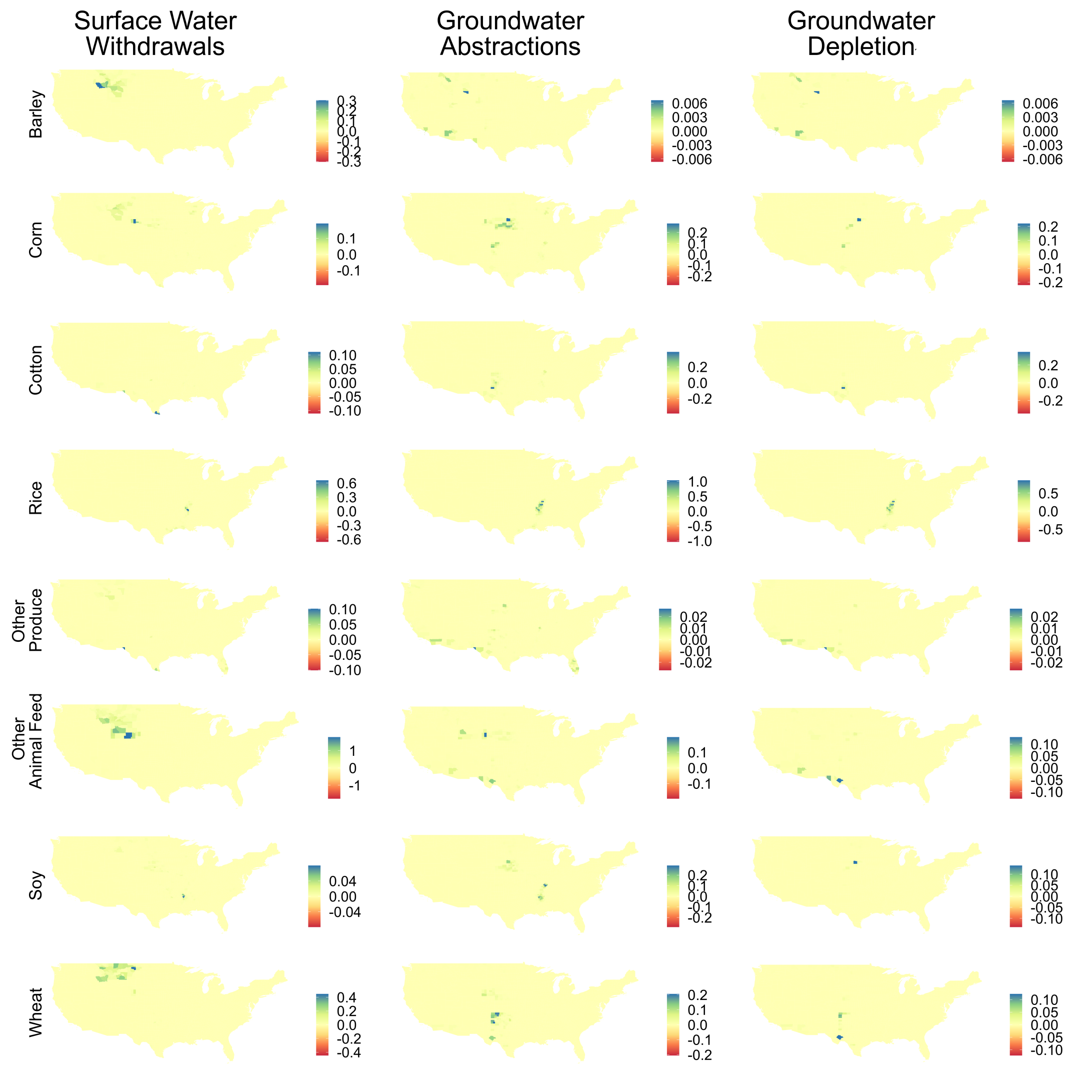

Figure 10: Maps of differences between Irrigation Water Use (IWU) modeled using Total Irrigation (from this work) vs. IWU modeled using Crop Irrigation (from Ruess et al. (2023)), organized by water source in 2017 (km^3 yr^{-1}). Columns show water source: Surface Water Withdrawals (SWW), Groundwater Withdrawals (GWW), and Groundwater Depletion (GWD). Rows show specific crops: barley, corn, cotton, rice, 'other produce', 'other animal feed', soy, and wheat.

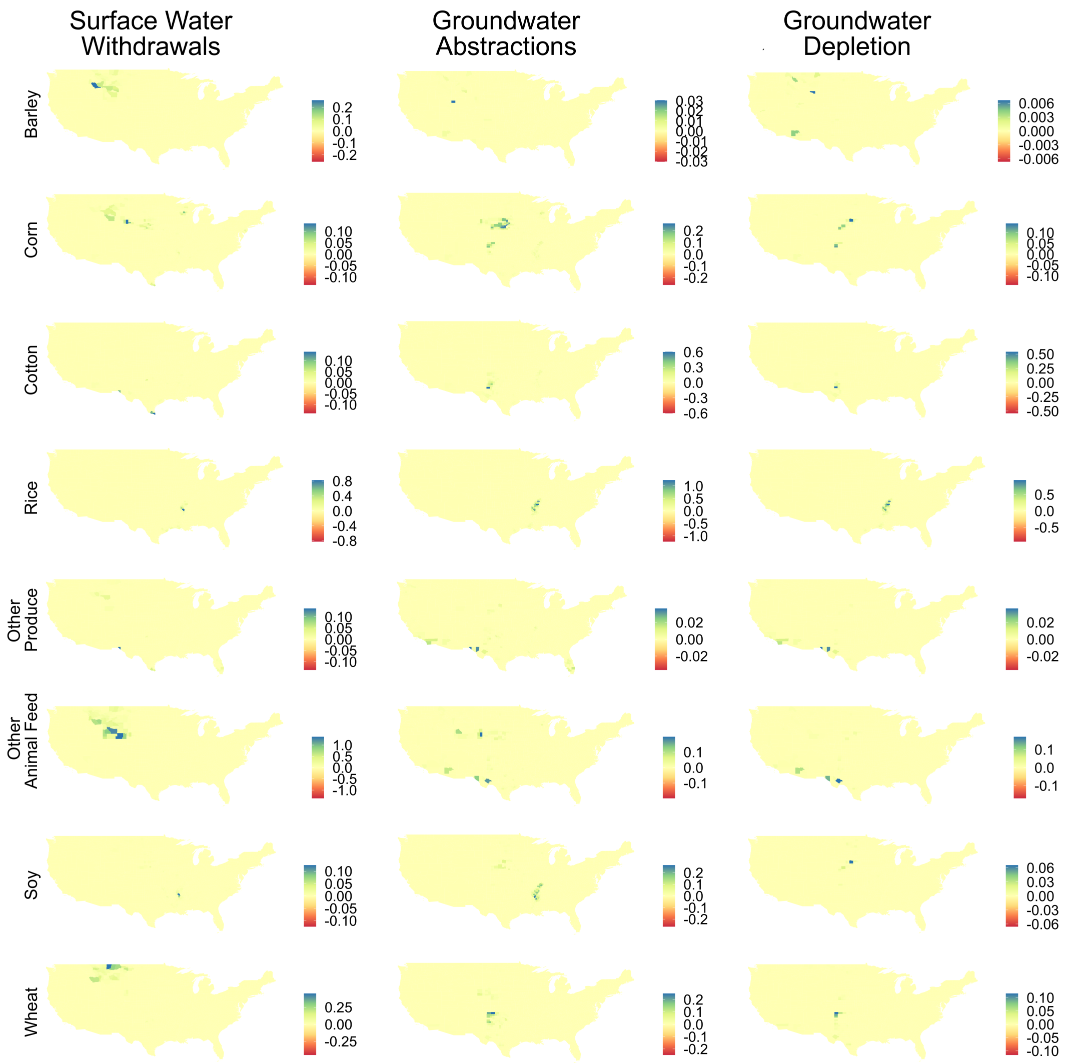

Figure 11: Maps of differences between Irrigation Water Use (IWU) modeled using Total Irrigation (from this work) vs. IWU modeled using Crop Irrigation (from Ruess et al. (2023)), organized by water source in 2018 (km^3 yr^{-1}). Columns show water source: Surface Water Withdrawals (SWW), Groundwater Withdrawals (GWW), and Groundwater Depletion (GWD). Rows show specific crops: barley, corn, cotton, rice, 'other produce', 'other animal feed', soy, and wheat.

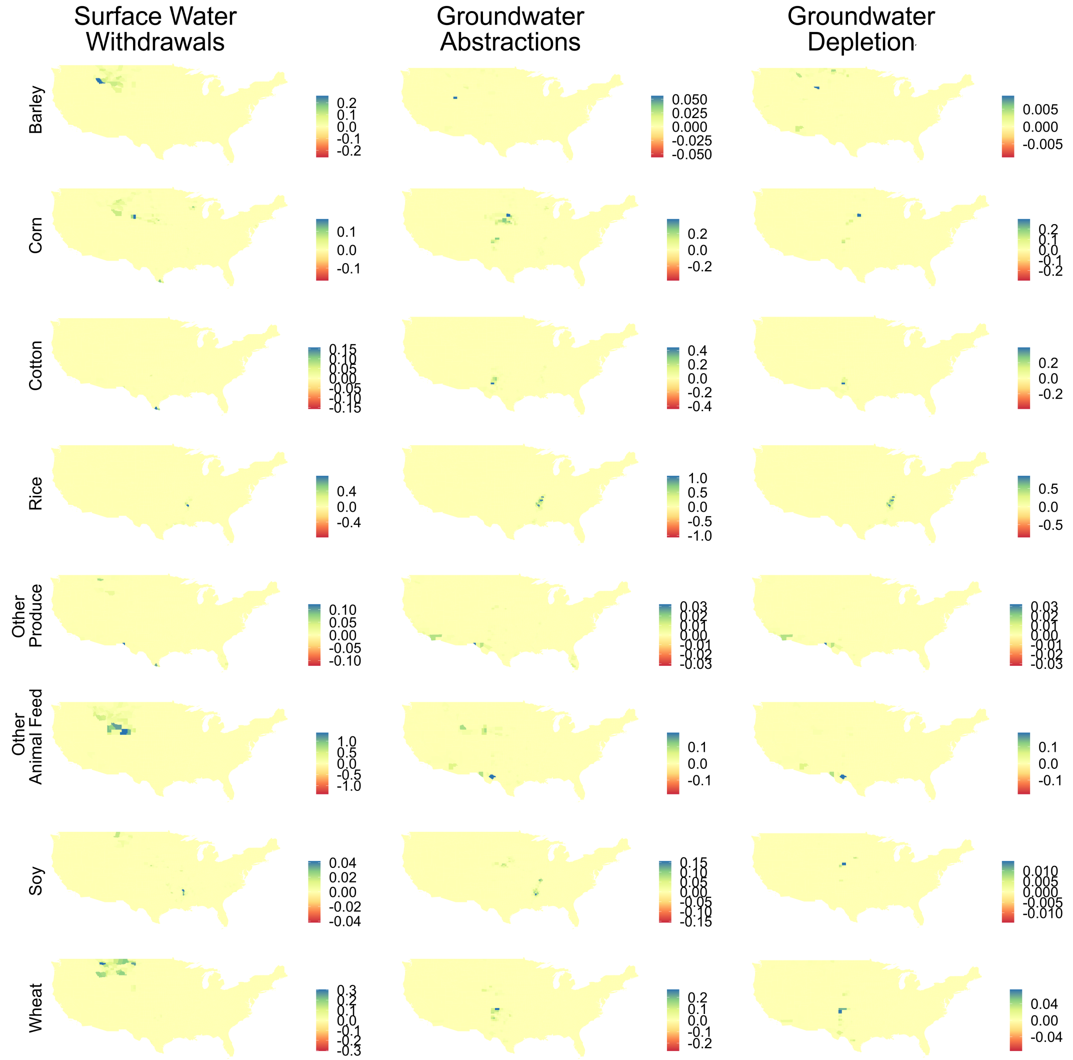

Figure 12: Maps of differences between Irrigation Water Use (IWU) modeled using Total Irrigation (from this work) vs. IWU modeled using Crop Irrigation (from Ruess et al. (2023)), organized by water source in 2019 (km^3 yr^{-1}). Columns show water source: Surface Water Withdrawals (SWW), Groundwater Withdrawals (GWW), and Groundwater Depletion (GWD). Rows show specific crops: barley, corn, cotton, rice, 'other produce', 'other animal feed', soy, and wheat.

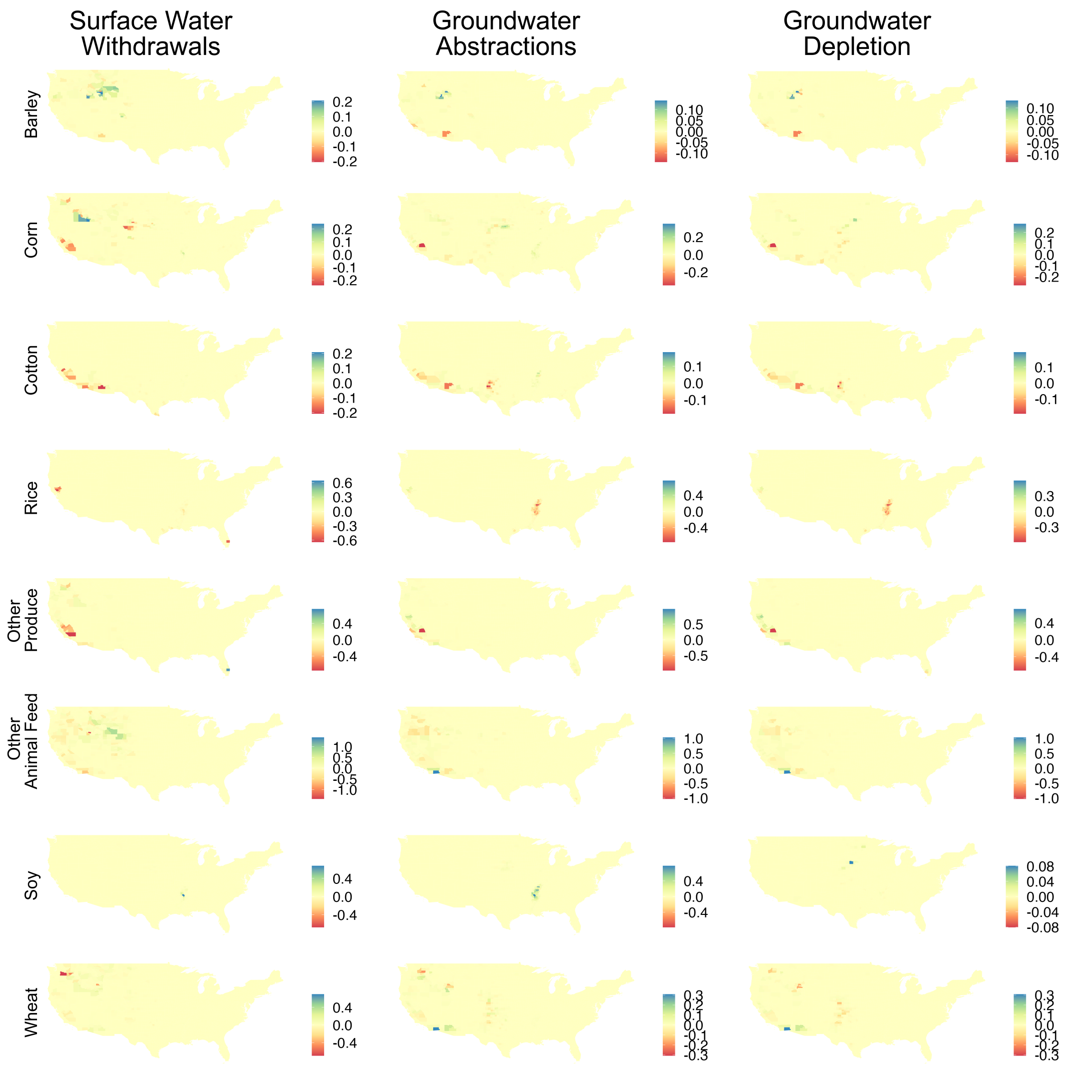

Figure 13: Maps of differences between Irrigation Water Use (IWU) modeled using Total Irrigation (from this work) in 2015 vs. IWU using Total Irrigation (from this work) in 2014, organized by water source (km^3 yr^{-1}). This plot is intended to show a change over time in IWU from 2014 to 2015 using the current model. Columns show water source: Surface Water Withdrawals (SWW), Groundwater Withdrawals (GWW), and Groundwater Depletion (GWD). Rows show specific crops: barley, corn, cotton, rice, 'other produce', 'other animal feed', soy, and wheat.

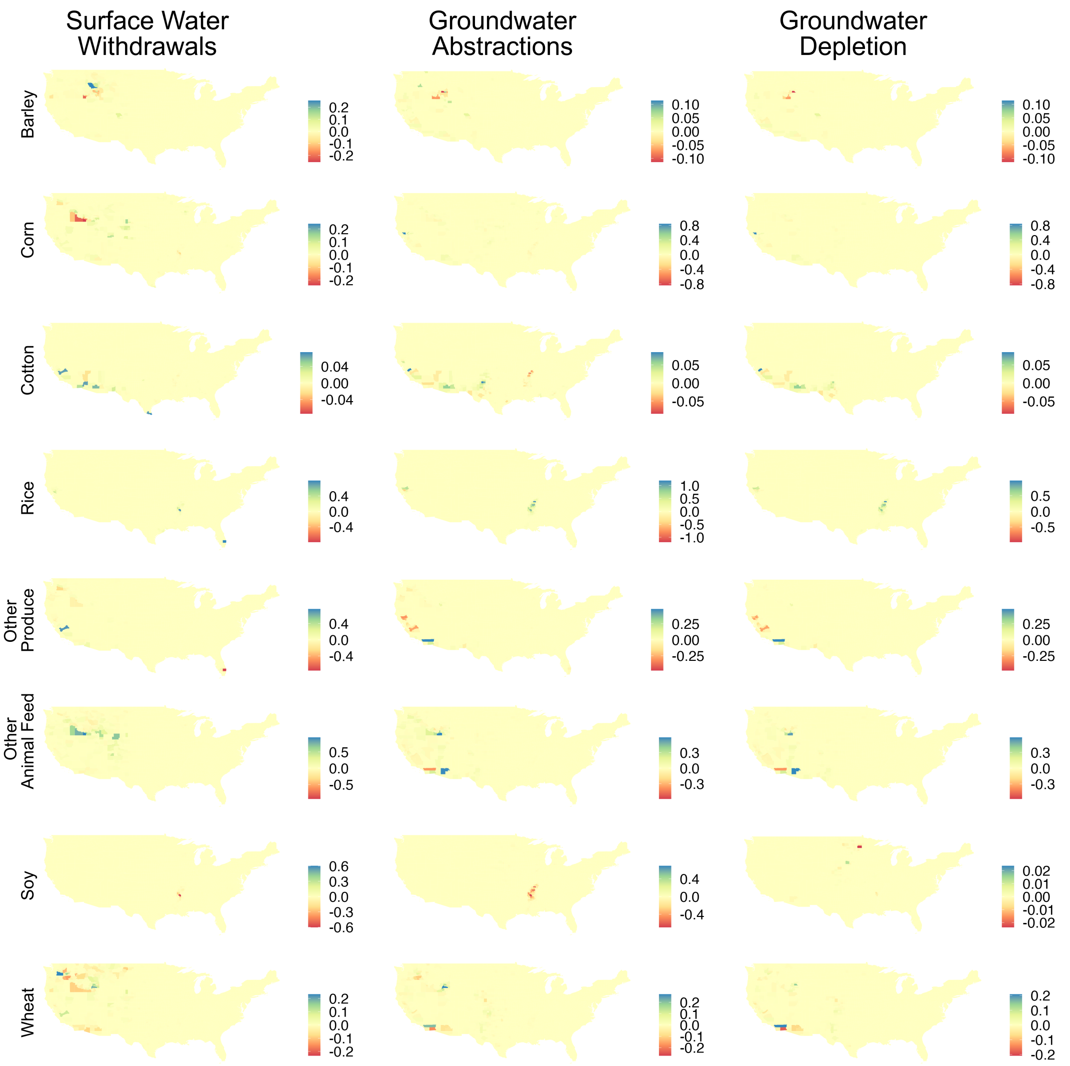

Figure 14: Maps of differences between Irrigation Water Use (IWU) modeled using Total Irrigation (from this work) in 2016 vs. IWU using Total Irrigation (from this work) in 2015, organized by water source (km^3 yr^{-1}). This plot is intended to show a change over time in IWU from 2015 to 2016 using the current model. Columns show water source: Surface Water Withdrawals (SWW), Groundwater Withdrawals (GWW), and Groundwater Depletion (GWD). Rows show specific crops: barley, corn, cotton, rice, 'other produce', 'other animal feed', soy, and wheat.

References

P. Ruess, M. Konar, N. Wanders, and M. Bierkens. Irrigation by Crop in the Continental United States From 2008 to 2020. *Water Resources Research*, 59(2), 2023. doi: 10.1029/2022WR032804.

USGS. Estimated Use of Water in the United States in 2015, 2018. URL `https://water.usgs.gov/watuse/data/data2015.html`.
